# Characterisation of the *Porphyromonas gingivalis* Manganese Transport Regulator Orthologue

**DOI:** 10.1371/journal.pone.0151407

**Published:** 2016-03-23

**Authors:** Lianyi Zhang, Catherine A. Butler, Hasnah S. G. Khan, Stuart G. Dashper, Christine A. Seers, Paul D. Veith, Jian-Guo Zhang, Eric C. Reynolds

**Affiliations:** 1 Oral Health Cooperative Research Centre, Melbourne Dental School, Bio21 Institute, The University of Melbourne, Melbourne, Victoria, Australia; 2 Walter and Eliza Hall Institute of Medical Research and Department of Medical Biology, The University of Melbourne, Melbourne, Victoria, Australia; Russian Academy of Sciences, Institute for Biological Instrumentation, RUSSIAN FEDERATION

## Abstract

PgMntR is a predicted member of the DtxR family of transcriptional repressors responsive to manganese in the anaerobic periodontal pathogen *Porphyromonas gingivalis*. Our bioinformatic analyses predicted that PgMntR had divalent metal binding site(s) with elements of both manganous and ferrous ion specificity and that PgMntR has unusual twin C-terminal FeoA domains. We produced recombinant PgMntR and four variants to probe the specificity of metal binding and its impact on protein structure and DNA binding. PgMntR dimerised in the absence of a divalent transition metal cation. PgMntR bound three Mn(II) per monomer with an overall dissociation constant *K*_d_ 2.0 x 10^−11^ M at pH 7.5. PgMntR also bound two Fe(II) with distinct binding affinities, *K*_d1_ 2.5 x 10^−10^ M and *K*_d2_ ≤ 6.0 x 10^−8^ M at pH 6.8. Two of the metal binding sites may form a binuclear centre with two bound Mn^2+^ being bridged by Cys108 but this centre provided only one site for Fe^2+^. Binding of Fe^2+^ or Mn^2+^ did not have a marked effect on the PgMntR secondary structure. Apo-PgMntR had a distinct affinity for the promoter region of the gene encoding the only known *P*. *gingivalis* manganese transporter, FB2. Mn^2+^ increased the DNA binding affinity of PgMntR whilst Fe^2+^ destabilised the protein-DNA complex *in vitro*. PgMntR did not bind the promoter DNA of the gene encoding the characterised iron transporter FB1. The C-terminal FeoA domain was shown to be essential for PgMntR structure/function, as its removal caused the introduction of an intramolecular disulfide bond and abolished the binding of Mn^2+^ and DNA. These data indicate that PgMntR is a novel member of the DtxR family that may function as a transcriptional repressor switch to specifically regulate manganese transport and homeostasis in an iron-dependent manner.

## Introduction

*Porphyromonas gingivalis* is a Gram-negative anaerobic bacterial pathogen strongly associated with chronic periodontitis; a chronic inflammatory disease of the supporting tissues of the teeth. Although *P*. *gingivalis* is a natural member of the oral microbiome, it can become highly destructive and proliferate to high cell numbers in periodontal lesions due to its arsenal of specialised virulence factors. *P*. *gingivalis* in the oral polymicrobial biofilm is a central player in the pathogenic potential of this microbial community through interactions with other species [1]. *P*. *gingivalis* requires iron and protoporphyrin IX as nutrients for growth, preferentially obtained in the form of haemoglobin-derived haem [2]. Although ferrous ion is essential for growth, it may be harmful if present in a free state as it may lead to generation of hydroxyl radicals [3, 4]. During colonization of the oral cavity, *P*. *gingivalis* is exposed to different environmental conditions, including altered nutrient availability, co-colonizing bacteria and the host response as well as various oxidative stress conditions created by reactive oxygen species. Therefore, in order to acquire nutrients from the host/environment for survival as well as protection against reactive oxygen species, *P*. *gingivalis* has to induce an efficient oxidative stress defence system as well as effective metal uptake systems by altering gene expression and subsequent protein production to overcome these conditions.

Mn(II) is needed by many bacteria for detoxification of reactive oxygen intermediates, as a cofactor for enzymes involved in metabolism, signal transduction and as a stimulus for virulence gene regulation [5–9]. Mn(II) may also be able to replace ferrous ions and rescue bacteria from Fe-induced stress [10]. *P*. *gingivalis* utilizes manganese for oxidative stress protection and intracellular survival in host cells [11, 12].

Encoded in the same operon as the only characterised manganese transporter in *P*. *gingivalis*, FB2, is PgMntR, a 35 kDa protein predicted to be a manganese-responsive member of the DtxR superfamily of transcriptional repressors [11]. DtxR family repressors are important for bacterial metal ion homeostasis, maintaining cytoplasmic metal concentrations in a desirable range for bacteria to grow [13, 14]. They bind two metal ions per monomer, which enables the dimeric repressor to bind DNA and prevent transcription of genes encoding proteins required for metal ion transport and homeostasis [15]. The DtxR family of iron-responsive metalloregulators form stable dimers in the presence of divalent metal cations, whereas the MntR subfamily of manganese-responsive regulators can form dimers in the absence of metal.

PgMntR has close sequence similarity to the DtxR family paradigms that function for both iron-responsive and manganese-responsive metalloregulation, namely *Corynebacterium diphtheriae* DtxR (CdDtxR) and *Bacillus subtilis* MntR (BsMntR) [15, 16], having elements of both Fe(II) and Mn(II) binding sites [11]. In order to elucidate the metal binding characteristics of PgMntR and the potential relationship of this to metal homeostasis in *P*. *gingivalis*, recombinant PgMntR and predicted primary metal binding site variants were prepared and the metal binding affinities and effects on the formation of PgMntR-DNA complexes were characterised. PgMntR also has two C-terminal FeoA domains which makes this protein longer than any previously characterised DtxR or MntR homologue, thus a PgMntR variant with the final C-terminal FeoA domain removed was created and the resulting effects on metal and DNA binding investigated.

## Materials and Methods

### Bioinformatic analyses

Sequence similarity searches were performed using the Basic Local Alignment Search Tool for protein sequences (BLASTp) program [17–19] at the National Centre for Biotechnology Information (NCBI) (http://blast.ncbi.nlm.nih.gov). To identify putative domains in the *P*. *gingivalis* W83 protein PG1044 (PgMntR), the primary sequence was subjected to a search via RPS-BLAST of the Conserved Domain Database at the NCBI website [20]. CLUSTAL Omega was used to perform alignments of multiple protein sequences through the European Bioinformatics Institute, part of the European Molecular Biology Laboratory (EMBL-EBI) (http://www.ebi.ac.uk/Tools/msa/clustalo/) [21, 22]. Metal binding sites of PgMntR were predicted based on the conserved amino acid residues which had been experimentally characterised as metal binding ligands in the protein homologues used in the analysis.

### Bacterial strains

*P*. *gingivalis* W50 was obtained from the culture collection of the Oral Health CRC, Melbourne Dental School, The University of Melbourne. *E*. *coli* BL21(DE3) was supplied by Novagen.

### Production of His-PgMntR wild-type and variant expression constructs

The 933 bp *pgmntR* (PG1044) open reading frame (ORF), and the 684 bp *pgmntR* ORF which encoded PgMntR without the C-terminal FeoA domain were PCR amplified (S1 Table) from the *P*. *gingivalis* W50 genome and cloned *Aat*II/*Sma*I into the multiple cloning site of pET47b (Novagen). This produced the plasmids pET47b-PgMntR and pET47b-PgMntR ΔFeoA2 that expressed PgMntR and the truncated ΔFeoA2 respectively with an N-terminal His-tag cleavable with the HRV 3C protease. Site-directed mutagenesis of full-length *pgmntR* was performed using the QuikChange Lightning Multi Site-Directed Mutagenesis Kit (Agilent) according to the manufacturer’s instructions, with the pET47b-PgMntR plasmid as template and the primers containing the mutated codons as listed in S2 Table. To achieve the D19M and the C108E single mutations, only one primer each (PgMntR D19M and PgMntR C108E respectively) was required, whereas to achieve the four mutations in one template required the concurrent use of 2 primers (PgMntR D19A and PgMntR C108A E111A H112A).

### Expression and purification

PgMntR and variants were expressed as N-terminally His-tagged proteins in *E*. *coli* BL21(DE3) transformed with pET47b plasmids harboring the full-length or FeoA truncated *pgmntR* and site-directed *pgmntR* mutants. The cells were grown in Luria-Bertani (LB) medium supplemented with kanamycin (30 μg/mL) at 37°C and when the OD_600_ was 0.8, the expression of His-tagged proteins was induced by the addition of IPTG to a final concentration of 1 mM and lasted for 5 h at 32°C. Upon harvesting, cells were lysed by sonication in 50 mM Tris·Cl, 500 mM NaCl, 1% Triton X-100 and EDTA-free protease inhibitors (cOmplete ULTRA Tablets, EDTA-free, Roche), pH 7.5. The clarified lysate was applied to a His affinity column (HisTrap FF, GE Health Care) in a binding buffer (50 mM Tris·Cl, 500 mM NaCl, 10 mM imidazole, pH 7.5) at 4°C. Bound proteins were eluted with a linear gradient of 0–300 mM imidazole after extensive washing with binding buffer. The His-tag was cleaved from the proteins by His-tagged HRV 3C protease (200 U/5 mg PgMntR) in TBS (50 mM Tris·Cl containing 150 mM NaCl, pH 7.5) and the cleaved His tag and HRV 3C protease removed by passing the enzymatic digests through a HisTrap column. Further purification was achieved using a cation- or an anion- exchange column (HiTrap SP or HiTrap Q, GE Healthcare) with a linear gradient of 0–600 mM NaCl in 20 mM phosphate buffer (pH 6.8) for protein elution followed by size-exclusion chromatography using a HiLoad 16/60 Superdex 200 prep column (GE Healthcare). Buffer exchange was conducted using an Econo-Pac^®^ 10DG Desalting column (Bio-Rad) when necessary. Target proteins were monitored by SDS-PAGE during purification. Protein concentration was determined using calculated extinction coefficients at 280 nm (S3 Table) spectroscopically on a UV-visible spectrometer (Varian Cary).

### In-gel enzymatic digestion and MALDI-MS

Purified recombinant PgMntR was resolved by SDS-PAGE and subjected to in-gel tryptic digestion as described previously [23]. Peptide mass fingerprinting analysis was carried out on an Ultraflex III MALDI (matrix-assisted laser dissociation ionization) TOF/TOF (time of flight) mass spectrometer (Bruker Daltonics) and proteins were identified using the Mascot v 2.2 search engine (Matrix Science, London, UK) [24].

### Electrospray ionization mass spectrometry (ESI-MS)

Protein molar masses were determined by ESI-MS on a quadrupole time-of-flight mass spectrometer (Agilent) as previously described [25].

### Inductively coupled plasma mass spectrometry (ICP-MS)

Metal contents of PgMntR and variants purified with and without treatment by the metal chelator EDTA were analysed by ICP-MS on an Agilent 7700 series instrument under routine multi-element operating conditions, using a Helium Reaction Gas Cell in the Analytical Service Facility at the Department of Pathology of the University of Melbourne as previously described [11].

### Analytical size exclusion chromatography (SEC)

Gel filtration analyses were performed at room temperature using an analytical Superdex 200 column and an AKTA FPLC chromatographic system (GE Healthcare) with buffers at pH 6.5 (20 mM MES) or pH 7.5 (50 mM Tris·Cl) each containing 150 mM NaCl. Protein molar masses were calculated against a calibration curve which was generated according to a gel filtration calibration kit (GE Healthcare).

### Sedimentation velocity-analytical ultracentrifugation (SV-AUC) analysis

Samples were analysed using an XL-I analytical ultracentrifuge (Beckman Coulter) equipped with an AnTi-60 rotor. PgMntR and variants at 10 or 60 μM were added to double-sector Epon-filled centerpieces, with TBS buffer (50 mM Tris·Cl, 150 mM NaCl, pH 7.5) in the reference compartment. Radial absorbance data was acquired at 20°C using a rotor speed of 40,000 rpm and a wavelength of 280 nm, with radial increments of 30 μm in continuous scanning mode. The sediment boundaries were fitted to a model that describes the sedimentation of species in solution with no assumption of heterogeneity (c(s)) using the program SEDFIT [26]. Data were fitted using a regularization parameter of p = 0.95, floating frictional ratios, and 150 sedimentation coefficient increments in the range of 0.1–20 S.

### Free thiol assay

Free sulfhydryl groups in PgMntR and variants were determined in air with Ellman’s reagent [27, 28] in sodium phosphate buffer (0.1 M, pH 8.0) and also under denaturing conditions (8 M urea in the same buffer) with DTT and glutathione (GSH) being used as controls.

### Fluorescence measurements and estimation of metal binding affinities

Fluorescence spectra were collected at room temperature on a Cary Varian spectrofluorometer. PgMntR and variants (5.0 μM) in either 50 mM Tris·Cl buffer (pH 7.5) or 50 mM HEPES (pH 6.8) each containing 150 mM NaCl were excited at 280 nm for collection of fluorescence spectra from 290 to 450 nm under non-reducing or reducing (2 mM Tris(2-carboxyethyl)phosphine, TCEP) conditions. During trial experiments to determine the metal binding stoichiometry of PgMntR by fluorescence titrations, PgMntR was found to aggregate upon addition of Mn^2+^ and Fe^2+^ at room temperature with visible precipitates starting from addition of around 0.3 molar equivalents of the metal ions. Therefore, a series of solutions with increasing metal:protein molar ratios (0–6) were prepared separately at 4°C under non-reducing (for Mn^2+^) and reducing (for either Mn^2+^ or Fe^2+^) conditions. A corresponding fluorescence spectrum of each solution was then collected. MnCl_2_ and (NH_4_)_2_Fe(SO_4_)_2_ were used as the metal ion sources. Metal binding affinities of the proteins were estimated from the fluorescence changes at 345 nm upon addition of corresponding metal ions. For those proteins with titration curves having distinct stoichiometric turning points due to a high saturation level of the metal binding sites, an upper limit of dissociation constants was estimated with an assumption of ≥ 90% of metal occupancy of the sites at the turning points. Metal binding affinities of the proteins were also estimated based on the fluorescence changes at 345 nm using ligand competition with the titration of the metal chelator EDTA at various metal ion:protein molar ratios. Blank titrations with EDTA alone were performed for corrections. Metal binding affinities were calculated as metal dissociation constants using the method of Zhang *et al* [25]. For those proteins without a distinct stoichiometric point in their titration curves, dissociation constants were estimated by fitting corresponding titration data sets using the biochemical analysis program Dynafit [29].

### Far UV circular dichroism spectroscopy

Circular dichroism (CD) measurements were performed on a Jasco J815 CD spectropolarimeter equipped with a thermostated cell holder and interfaced with a Peltier unit. Protein samples were prepared at 4°C. Spectra were collected from 260 to 190 nm with the CD signal being recorded in a 0.1 cm path length Helma quartz cuvette. CD spectra of each protein (5.0 μM) were collected with averaging over three accumulation scans in the absence or presence of Mn^2+^ or Fe^2+^ in MES (5 mM, pH 6.5) or Tris·Cl (5 mM, pH 7.5) buffer containing 15 mM NaCl under reducing (2 mM TCEP) or non-reducing conditions. Secondary structures were estimated by deconvolution of the spectra via the online DichroWeb CD analysis program [30, 31].

### Electrophoresis mobility shift assay (EMSA)

Oligonucleotide primers (Geneworks) were designed to PCR amplify 385 bp which encompassed the promoter region upstream of the *pgmntR* gene (P1; *P*. *gingivalis* W83 gDNA nt 1,113,909–1,114,293) and 272 bp upstream of the *feoB1* gene (FB1p; *P*. *gingivalis* W83 gDNA nt 1,371,915–1,372,186 [32]). A 385 bp internal region of the PG1656 gene (C1; *P*. *gingivalis* W83 gDNA nt 1,737,949–1,738,333 [32]) was amplified for use as a negative control in the EMSA. Biotinylated amplicons were produced using primers containing 5’ biotin groups (S4 Table). All PCR products were purified using the NucleoSpin^®^ Extract II purification kit (Macherey Nagel) according to manufacturer’s instructions. Binding of PgMntR and variants to DNA was examined using the LightShift^®^ Chemiluminescent EMSA kit (Thermo Scientific) according to manufacturer’s instructions. Briefly, PgMntR and variant proteins (700–1000 nM) were incubated with the biotinylated DNA (1 nM) in the presence or absence of Mn^2+^ (0–200 μM) and Fe^2+^ (0–40 μM) in a 10 μL solution of 90 mM Bis-Tris borate (pH 6.8), 10 mM Tris·Cl (pH 7.5) or Tris-Acetate (pH 6.8) and 50 mM KCl containing 2.5% glycerol, 0.05% NP-40 and 50 ng/μL non-specific DNA competitor poly(dI-dC). Reducing conditions in the binding reactions were maintained by 1 mM TCEP and 5 mM DTT. Up to 1 mM EDTA was included in the binding reactions where appropriate for investigation of apo-PgMntR binding to DNA. After incubation for 30 min at room temperature, the samples were subjected to electrophoresis through a 4% non-denaturing polyacrylamide mini-gel in 45 mM Bis-Tris borate (pH 6.8), 0.5 x TBE (pH 7.6) or 0.5 x TAE (pH 6.8) for 100 min at a constant 100 V. The DNA and protein complexes were then transferred to a positively charged nylon membrane and detected using the LightShift^®^ Chemiluminescent EMSA kit as per the manufacturer’s instructions. The complex formation was quantitated by measuring the unbound DNA band intensity with ImageQuant^™^ TL analysis software (GE Heathcare). A recombinant dimeric transcriptional regulator Har from *P*. *gingivalis* that binds DNA and regulates haem-responsive biofilm formation [33] was used as a negative control protein.

Where appropriate, labware and buffers used in this study were treated with 20% HNO_3_ and Chelex 100 respectively. All solvents and buffers were free of divalent metal cations which were confirmed to be below or at the detection limits of ICP-MS.

## Results

### Bioinformatic analyses

**Fig 1:**
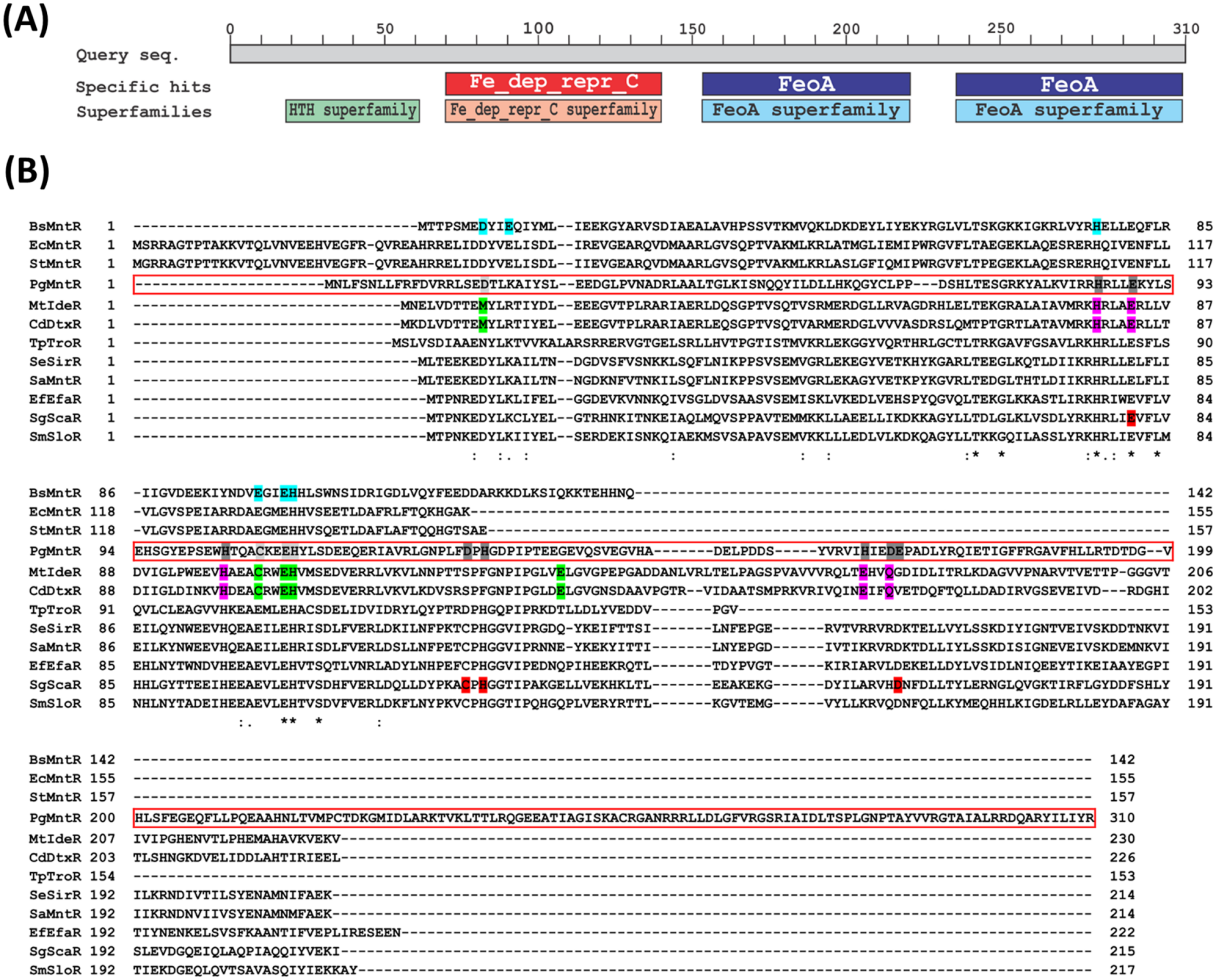
Examination of the PgMntR sequence. (A) The PgMntR sequence was used to search the Conserved Domain Database through NCBI using default parameters [20]. The four conserved domains of PgMntR are shown. (B) Clustal Omega with default parameters was used to align PgMntR with eleven experimentally characterised DtxR family homologues for the identification of potential metal binding amino acid residues. These homologues include the three iron-responsive transcriptional regulators, namely DtxR from *Corynebacterium diphtheria* (CdDtxR), IdeR from *Mycobacterium tuberculosis* (MtIdeR) and SirR from *Staphylococcus epidermidis* (SeSirR). The other eight are manganese-responsive transcriptional regulators, MntR from *Bacillus subtilis* (BsMntR), ScaR from *Streptococcus gordonii* (SgMntR), MntR from *E*. *coli* (EcMntR), TroR from *Treponema pallidum* (TpTroR), SloR from *Streptococcus mutans* (SmSloR), EfaR from *Enterococcus faecalis* (EfEfaR), MntR from *Staphylococcus aureus* (SaMntR) and MntR from *Salmonella enterica* Serovar Typhimurium (StMntR). The residues of the primary and secondary metal binding sites in CdDtxR and MtIdeR are shaded green and magenta respectively. Residues of the secondary metal binding site in SgScaR are shaded red as the primary binding site was not detected. The six residues that form the binuclear manganese binding centre in BsMntR are shaded aqua. Residues that are predicted to constitute the primary metal binding site in PgMntR are shaded light gray, whilst further potential metal binding residues are shaded dark gray. An asterisk (*) below the alignment indicates a fully conserved residue, whereas a colon (:) indicates conservation between groups of strongly similar properties (scoring > 0.5 in the Gonnet PAM 250 matrix) whilst a period (.) indicates conservation between groups with weakly similar properties (scoring = < 0.5 in the Gonnet PAM 250 matrix) [21, 22].

PgMntR has a similar three domain structure to the well characterised *C*. *diphtheriae* DtxR (CdDtxR); a predicted N-terminal DNA binding domain (aa 13–65), a metal-binding and dimerization domain (aa 71–141) and a C-terminal FeoA domain (aa 151–222). However a search using the PgMntR sequence against the NCBI conserved domain database revealed the presence of a second FeoA domain (aa 236–310) which has not previously been described (Fig 1A). Potential PgMntR homologues with significant similarity to the entire sequence of PgMntR were identified using BLASTp. There were 41 significant matches, representative of 13 bacterial genera from three different phyla that had significant sequence similarity to ≥ 93% of the PgMntR sequence, suggesting that these putative proteins are also DtxR-like with two FeoA domains (S1 Fig). However none of these putative proteins have been experimentally characterised. PgMntR also has sequence similarity with experimentally characterised DtxR superfamily proteins such as CdDtxR which has 45% similarity to 72% of the PgMntR sequence from the N-terminus, as CdDtxR does not have the 2^nd^ FeoA domain found in PgMntR. Thus PgMntR was aligned with experimentally characterised DtxR superfamily proteins in an attempt to predict PgMntR residues involved in metal binding (Fig 1B). From this comparison PgMntR residues Asp19, Cys108, Glu111 and His112 were predicted to constitute one metal binding site, while His85, Glu89, His104, Asp132, His134, His166, Asp169 and Glu170 were identified as further potential metal binding residues (Fig 1B). The four residues of the predicted metal binding site were aligned with the ligands in both the conserved iron-specific binding site (Met10, Cys102, Glu105 and His106) in CdDtxR and the manganese-specific binding site (Asp8, Glu99, Glu102 and His103) in the homologue BsMntR. Thus PgMntR is predicted to have both iron- and manganese-binding elements. These four residues in PgMntR were targeted for examination via mutagenesis, with Asp19 being changed to Met and Cys108 being changed to Glu for generation of an Fe^2+^-specific or a Mn^2+^-specific site respectively. All 4 residues; Asp19, Cys108, Glu111 and His112, were replaced with Ala to disable the site. In addition, a PgMntR variant, ΔFeoA2, without the final C-terminal FeoA domain, was prepared to investigate the effects of this additional FeoA domain on the metal and DNA binding.

### Expression and purification of recombinant wild-type PgMntR and its mutated variants

**Fig 2:**
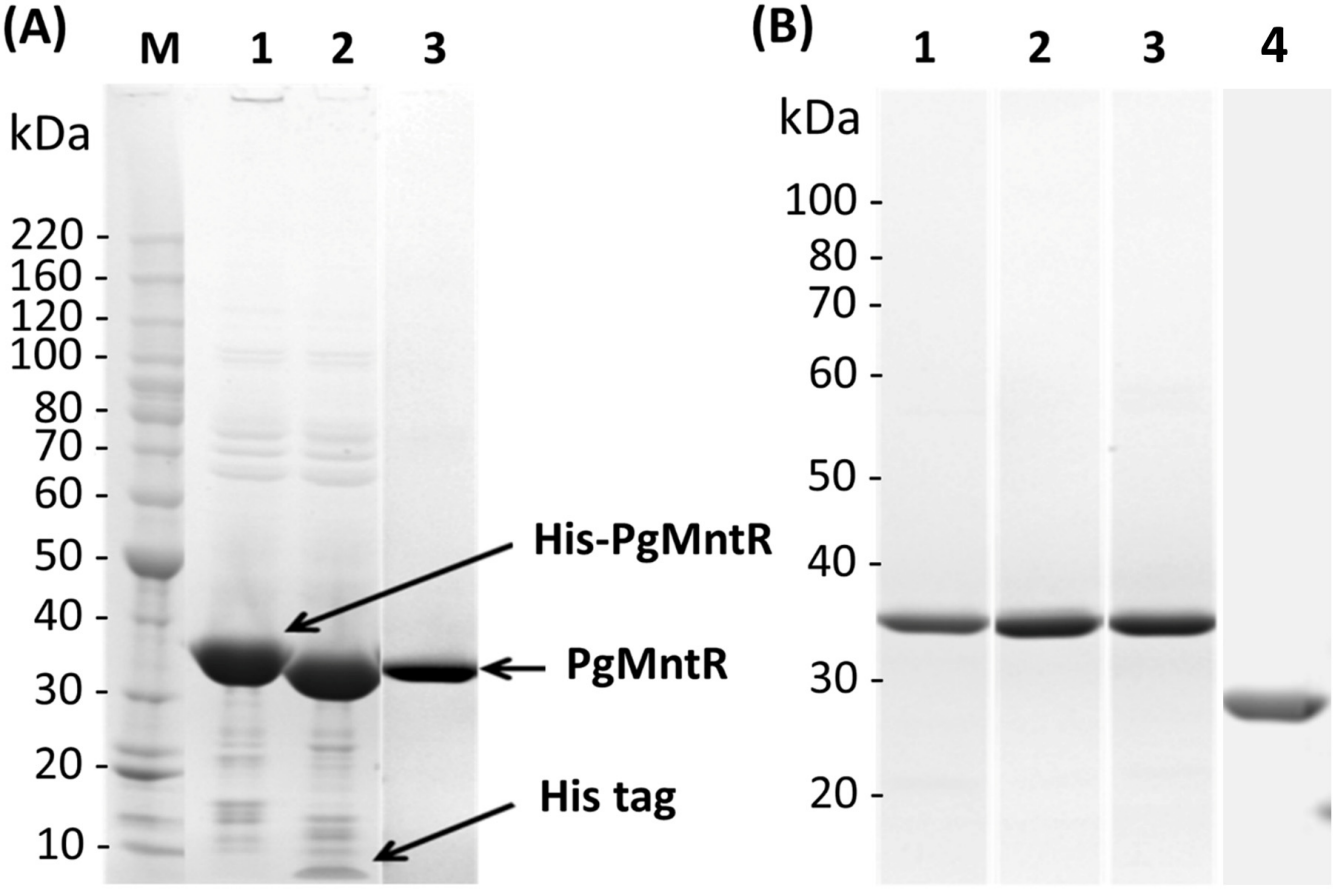
SDS-PAGE analysis of recombinant PgMntR and variants. (**A**) Lane M: BenchMark protein standard (Life Technologies); Lane 1: His-tagged PgMntR before cleavage with the HRV 3C protease; Lane 2: PgMntR after the His tag was cleaved; Lane 3: Purified PgMntR after nickel affinity, ion exchange and size-exclusion chromatographic steps. **(B)** Purified PgMntR variants D19M (Lane 1), C108E (Lane 2), 4Ala (Lane 3) and ΔFeoA2 (Lane 4). Proteins were resolved using 4–12% Bis-Tris polyacrylamide gels and were stained with Coomassie blue G. The identity of the His-tag shown in (**A**) was determined using MALDI-TOF peptide mass fingerprinting.

PgMntR and the four variants D19M, C108E, 4Ala (D19A/C108A/E111A/H112A) and ΔFeoA2 were expressed in *E*. *coli* using the pET His-tag fusion system and enriched via HisTrap affinity chromatography. The His-tags were cleaved in-solution by the HRV 3C protease, and removed together with the protease, via a second round of HisTrap affinity chromatography. Further purification by ion exchange and size exclusion chromatography removed some minor degradation products that were determined via trypsin in-gel digestion and MALDI-MS to be fragments of PgMntR (data not shown). Final protein products reached a purity of over 95% with a yield of ca. 5 mg/L culture (Fig 2). No His-tagged PgMntR or PgMntR variants were detected in the final purified products by immunoblotting using a monoclonal antibody against the polyhistidine tag (S2 Fig). The molar mass of each product as determined by ESI-MS closely matched the expected mass of each product free of His tags (S3 Table). In the purified PgMntR and variant proteins, no bound transition metals including Mn, Zn, Fe, Co, Cu, Ni and Cr were detected using ICP-MS, whether they were purified in the presence or absence of EDTA (S5 Table). PgMntR was highly soluble in TBS buffer at pH 7.5, reaching a concentration up to 50 mg/mL. PgMntR, which has a calculated isoelectric point (pI) of 5.77, precipitated at pH values between 5.0–6.0. Notwithstanding this predicted acidic pI PgMntR did not bind to an anion-exchange column at neutral pH but bound to a cation-exchange column, possibly via the 2^nd^ FeoA domain that has a predicted pI of 11.36. This was supported by the ability of the ΔFeoA2 protein, with a lower predicted pI (4.93) to bind to an anion-exchange column at neutral pH whilst the other three point-mutated variants with the retained second FeoA domain bound to a cation-exchange column. This result is consistent with the positive charges on the second FeoA domain being surface exposed.

### Oligomeric states of PgMntR proteins

Recombinant PgMntR and the variants D19M, C108E and 4Ala were analysed by SV-AUC to assess their oligomeric states. Each gave a predominantly single peak in the size distribution plot with a modal sedimentation coefficient of approximately 4S (S3 Fig). Taking into account this value and fitting the frictional ratios gave estimated molar masses of 63±7 kDa, suggesting that each protein existed as a dimer (S6 Table). The presence of Mn^2+^ did not affect the dimeric state of these proteins (S6 Table). The inclusion of the reducing agent TCEP did not dissociate the dimer of PgMntR indicating dimer formation was not due to intermolecular disulfide bonds (S6 Table). Elution profiles of PgMntR, D19M, C108E or 4Ala when analysed by size-exclusion chromatography were consistent with the SV-AUC results (S3 Fig). ΔFeoA2 was also determined to be a dimer independent of the presence of Mn^2+^ (S6 Table).

### Free thiol groups of PgMntR

There are a total of four cysteines (Cys63, Cys108, Cys223 and Cys256) in PgMntR. Only three thiols were detected in PgMntR with Ellman’s reagent under non-denaturing, non-reducing conditions. Under denaturing conditions in 8 M urea, 3.7 thiols were detected (Table 1), suggesting that one cysteine was buried in the folded protein sterically preventing access of its thiol group to Ellman’s reagent. This was also the case for variant D19M. Similarly, in the two other PgMntR variants, (C108E and 4Ala where Cys108 was replaced, leaving three cysteines), two thiol groups were detected under non-denaturing conditions whereas three thiol groups were detected under denaturing conditions. The free thiol assay conclusively established that the PgMntR dimer was not disulfide-linked. The ΔFeoA2 variant also has three Cys residues but only one third of the total present were detected under non-denaturing conditions and two thirds were detected under denaturing conditions (Table 1), suggesting that the final one third of the total Cys residues present were involved in disulphide bonds. Since no disulfide bridged dimers of ΔFeoA2 were detected using ESI-MS (S3 Table), the disulfide bond must have been intramolecular. Thus the truncation of the second FeoA domain affected the conformation of PgMntR allowing some cysteines to interact within the protein molecule to form a disulphide bond.

**Table 1:** Free thiol groups in PgMntR and variants determined using Ellman’s reagent (DTNB) under non-denaturing and denaturing (8 M urea) conditions with GSH and DTT as controls.

Condition	PgMntR^a^	D19M	C108E	4Ala	ΔFeoA2	GSH	DTT
nondenatured	3.0^b^	2.7	1.9	2.0	1.0	1.0	2.0
denatured	3.7	3.9	2.8	2.8	1.9	1.0	2.1

^a^PgMntR and D19M have a total of four cysteine residues whilst C108E, 4Ala and ΔFeoA2 have three. GSH and DTT have one and two free sulfhydryl groups respectively.

^b^Mean of three independent determinations.

### Metal binding stoichiometry and affinity

**Fig 3:**
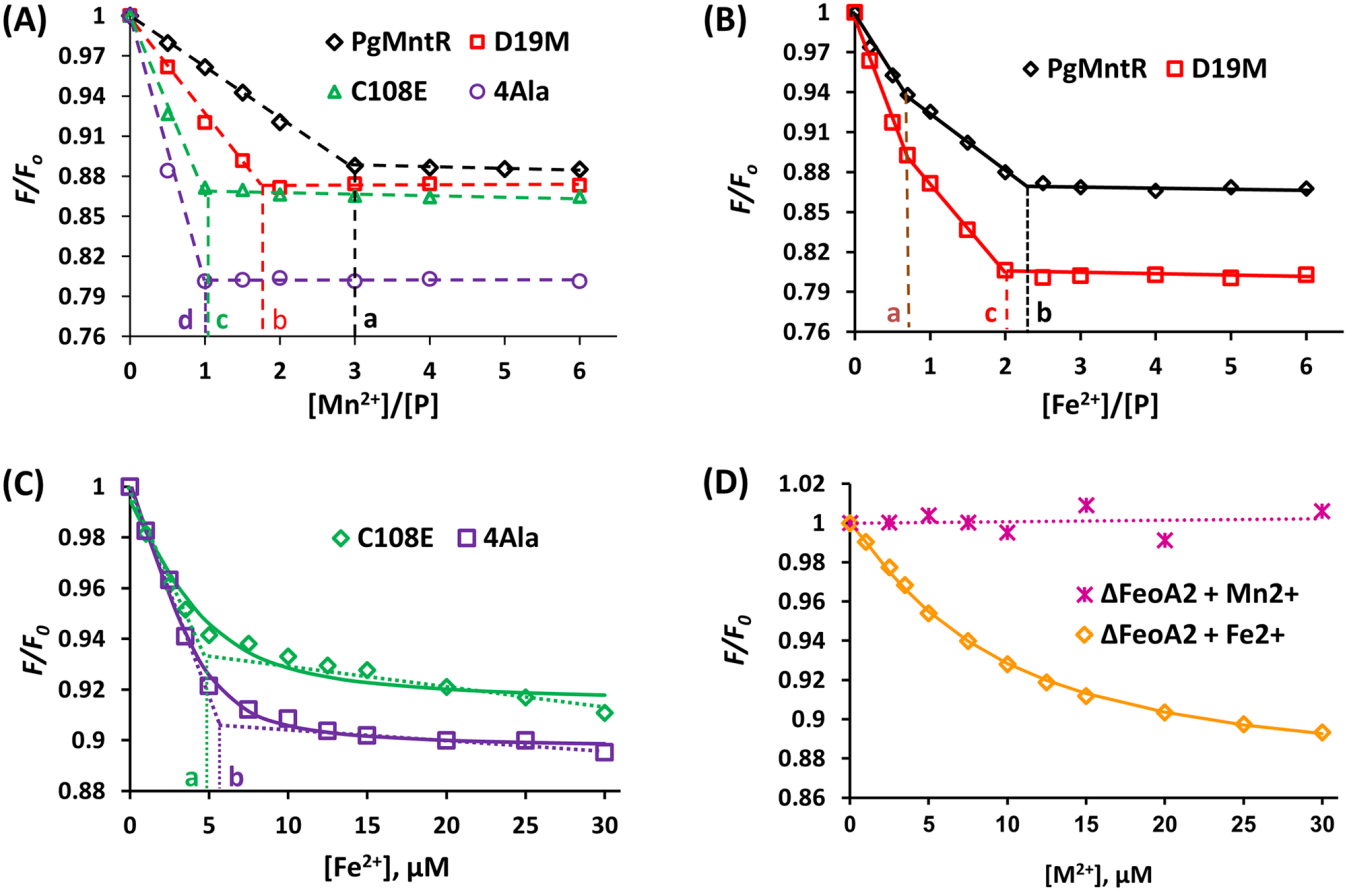
Manganous and ferrous ion binding by recombinant PgMntR and variants. Titration curves are shown as fluorescence changes at 345 nm (normalised as *F/F*_*0*_) of the PgMntR and variant proteins (5.0 μM) upon addition of Mn^2+^ under non-reducing conditions (**A and D**) and Fe^2+^ under reducing conditions (**B**, **C and D**). The corresponding stoichiometric reaction end points projected on the X-axis are indicated by a-d (as in metal to protein molar ratios in **A** and **B** and in added metal concentrations in **C**). As there are no clean turning points in the Fe^2+^ titration curves for C108E and 4Ala (**C**), the stoichiometric points are estimated by linear extrapolation from both ends of the titration curves (dotted lines). Solid lines in (**C and D**) are the curves generated from fitting using the biochemical analysis program Dynafit with the reaction stoichiometry of 1:1 metal to protein ratio[29]. Non-reducing buffer: TBS, pH 7.5; Reducing buffer: 50 mM HEPES, 150 mM NaCl, 2 mM TCEP, pH 6.8. M^2+^ = Mn^2+^ or Fe^2+^. *λ*_ex_ = 280 nm.

PgMntR and its variants exhibited intrinsic fluorescence emission with a maximum intensity at 345 nm upon excitation at 280 nm owing to a tryptophan residue (Trp103) which acts as a fluorophore. Fluorescence emission intensity at 345 nm (*F*_345_) was quenched upon addition of Mn^2+^ under reducing and non-reducing conditions or by Fe^2+^ under reducing conditions, indicative of metal-protein interactions for all proteins except ΔFeoA2 with Mn^2+^ (Fig 3). Under non-reducing conditions at pH 7.5, addition of Mn^2+^ to PgMntR quenched *F*_345_ linearly up until three molar equivalents of Mn^2+^ after which quenching leveled off, suggesting the protein bound three manganous ions per monomer with high affinity (Fig 3A). The variants also exhibited linear decreases in fluorescence, but flattened at different stoichiometries. D19M bound two Mn(II) per monomer, whilst C108E and the quadruple mutant 4Ala only bound a single Mn(II) (Fig 3A).

In contrast to these variants, the fluorescence of ΔFeoA2 remained steady following additions of Mn^2+^ up to 6.0 molar equivalents suggesting that the additional C-terminal FeoA domain was essential to allow Mn(II) binding to any of the three sites (Fig 3D). PgMntR retained its binding activity of three Mn(II) per monomer under reducing conditions in the presence of 2 mM TCEP at pH 6.8 (data not shown).

When titrated against ferrous ions, the fluorescence of PgMntR and D19M was quenched up to a stoichiometry of two Fe(II) per monomer under reducing conditions at pH 6.8, however the quenching trace did not fit to a single linear relationship **(**Fig 3B). Global curve fitting of the data was unsuccessful (standard error as high as 10 times the calculated dissociation constants). Instead, the best fit for the data was two linear relationships with the first distinct turning point at a metal to protein molar ratio of 0.75 and a second turning point at a ratio of approximately 2.0 (Fig 3B). This suggests that there are two separate iron binding sites with significantly different binding affinities for Fe^2+^. The two sites, one with high affinity (Site H) and the other with much lower affinity (Site L) were sequentially occupied by Fe(II) with the continued addition of Fe^2+^. The establishment of a clear stoichiometric turning point at the 2:1 Fe:PgMntR molar ratio and no further quenching of fluorescence after this turning point (Fig 3B) suggested that both Site H and Site L were fully occupied by Fe(II). Assuming that at least 90% of Site L was occupied whilst the amount of Site H unoccupied by metal ions was negligible at this reaction end point, the Fe(II) affinity of Site L was estimated to have a dissociation constant K_dL_ ≤ 6.0 x 10^−8^ M while K_dH_ << K_dL_. Similarly, D19M also had the dissociation constants in the range of K_dH_ << K_dL_ ≤ 6.0 x 10^−8^ M.

Titration of Fe^2+^ into two different concentrations of C108E or 4Ala (1.0 and 5.0 μM) did not result in a clear turning point as for PgMntR or D19M (Fig 3C and S4 Fig), suggesting the existence of an iron binding equilibrium. Linear extrapolation of the data suggested that C108E and 4Ala only bound one ferrous ion each. Global curve-fitting of the data was found to give a close fit and provided an apparent K_d_ = 5.0 x 10^−7^ (± 1.0 x 10^−7^) M for C108E and a K_d_ = 3.0 x 10^−7^ (± 1.0 x 10^−7^) M for 4Ala at total protein concentrations of both 1.0 and 5.0 μM (Fig 3C and S4 Fig). Therefore, replacement of Cys108 by either Glu or Ala resulted in not only the loss of a Fe(II) binding site but also a decrease in Fe(II) binding affinity of the remaining site, suggesting a crucial role of Cys108 in Fe(II) binding by PgMntR (Fig 3B and 3C). Similarly, titration of Fe^2+^ into 5.0 μM ΔFeoA2 also did not produce a clear turning point as for PgMntR (Fig 3D). Dynafit fitting estimated a K_d_ = 6.1 x 10^−6^ (± 0.5 x 10^−6^) M for FeII) binding by ΔFeoA2, 12 or 20 times weaker than that of C108E or 4Ala respectively.

Addition of 15 molar equivalents of EDTA restored the fluorescence intensity of the solutions of PgMnR which had been quenched by addition of 3 equivalents of Mn^2+^ or Fe^2+^, confirming the bound state of PgMntR with either of the two metals (data not shown). Ligand competition equilibrium analysis with the metal chelator EDTA was used to determine more accurate metal binding affinities of PgMntR for Mn(II) and Site H for Fe(II). To determine the Fe^2+^ binding affinity for Site H of PgMntR, 0.5 molar equivalent Fe^2+^ was used so that the lower affinity Fe(II) binding site (Site L) was vacant. Upon addition of 0.5 molar equivalent Fe^2+^ to PgMntR, fluorescence of the protein was quenched corresponding to the expected 50% occupancy of Fe(II) Site H. The titration addition of the competitor EDTA incrementally restored fluorescence. For example, addition of 0.5 and 1.0 equivalents of EDTA restored fluorescence by 74% and 93%, corresponding to 14% and 4.2% occupancy of the higher affinity site by ferrous ion, respectively. Based on the EDTA competition data the apparent dissociation constant of Site H at pH 6.8 was estimated to be K_dH_ 2.5×10^−10^ M using the EDTA-Fe(II) dissociation constant of 1.6×10^−11^ M and acidity coefficient α(6.8)[EDTA] of 3.2×10^−4^ at pH 6.8 [34]. With the same approach, the apparent dissociation constant of the higher affinity Site H in D19M at pH 6.8 was determined to be K_dH_(D19M-Fe(II)) 2.5×10^−11^ M, one order of magnitude lower than that of PgMntR. Therefore, the substitution of Met for Asp19 increased the binding affinity of PgMntR for ferrous ion which can be attributed to the change to the more Fe(II) favourable ligand Met.

When the three Mn(II) binding sites with close binding affinities were treated as a combined Mn(II) site with one overall binding affinity, ligand competition between EDTA and PgMntR at the different EDTA:protein molar ratios estimated the apparent Mn(II) binding affinity to be *K*_d_ 2.0×10^−11^ M, based on the EDTA-Mn(II) dissociation constant of 6.3×10^−12^ M and acidity coefficient α_(7.5)_[EDTA] of 2.0×10^−3^ at pH 7.5 [34].

### Secondary structure of PgMntR in the presence and absence of Mn^2+^ or Fe^2+^

**Fig 4:**
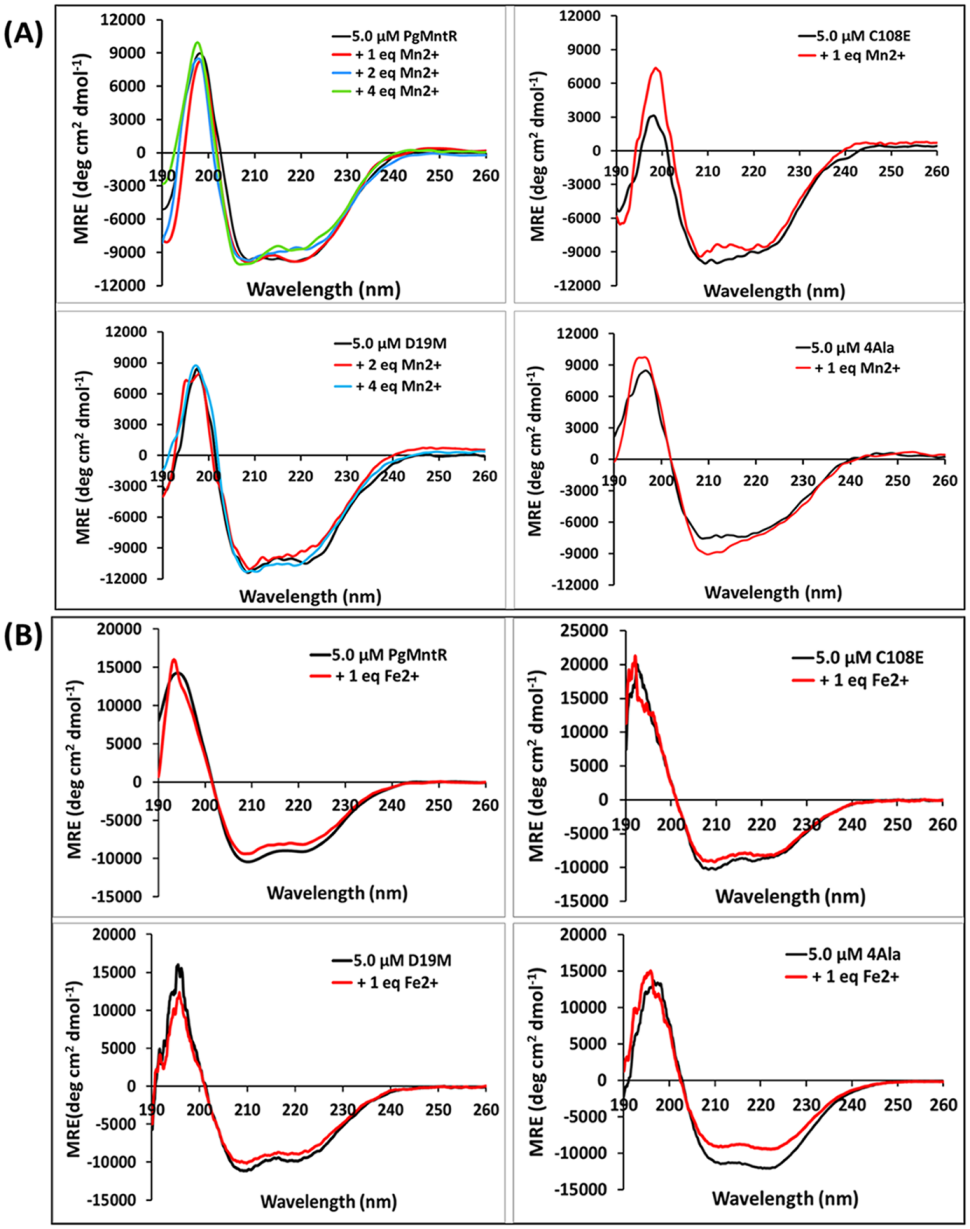
CD spectra of PgMntR and variants D19M, C108E and 4Ala in the presence and absence of Mn^2+^ or Fe^2+^. (A) In the presence or absence of 1–4 molar equivalents of Mn^2+^ at pH 7.5 in 5 mM Tris∙Cl containing 15 mM NaCl. (B) In the presence or absence of one molar equivalent of ferrous ions at pH 6.5 in 5 mM MES containing 15 mM NaCl. MRE: mean residue ellipticity.

Deconvolution of the CD data (Fig 4) suggested that the secondary structure of apo-PgMntR consists of around 35–40% α-helices, 15–20% β-strands and 40–45% others, with no significant changes upon addition of manganous or ferrous ions. Similarly, CD did not detect significant changes in the secondary structure composition of the variants D19M, C108E, 4Ala and ΔFeoA2 upon addition of the metal ions (Fig 4 and S5 Fig). No marked changes were detected in the relative percentages of the secondary structure components caused by the truncation of the additional FeoA domain.

### PgMntR-DNA binding

**Fig 5:**
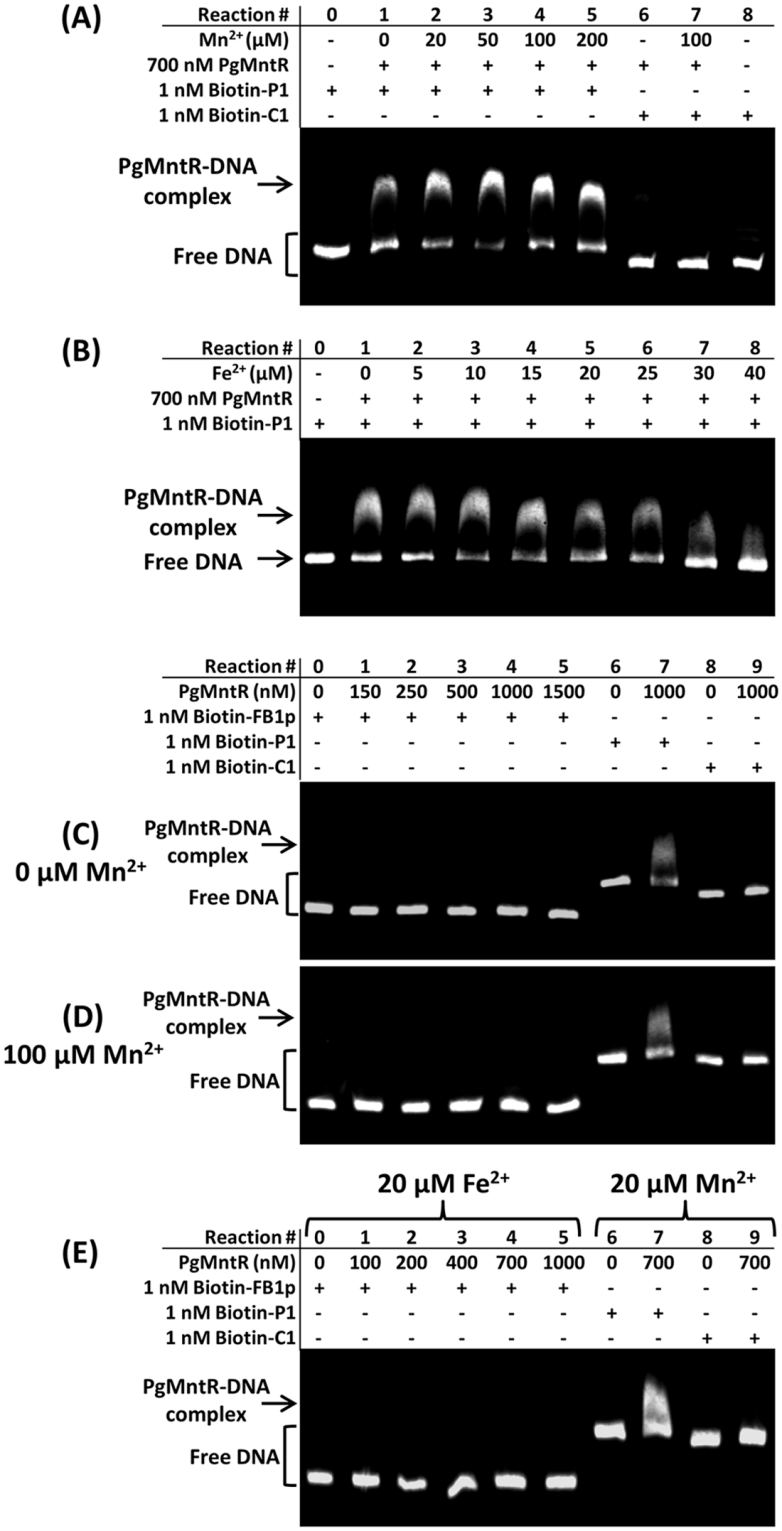
EMSA of PgMntR binding to the promoter sequences upstream of the *pgmntR* gene (P1) and the *feoB1* gene (FB1p). (A) Binding to P1 in the presence or absence of Mn^2+^, with PG1656 (C1) as a negative control. (B) Binding to P1 at varied concentrations of Fe^2+^. (C)—(E) Binding to FB1p in the presence or absence of Mn^2+^ or Fe^2+^, with C1 and P1 as negative and positive controls, respectively. All reactions were performed in a 10 μL solution buffered with 90 mM Bis-Tris borate (pH 6.8) containing 50 mM KCl, 1 mM TCEP, 5 mM DTT, 2.5% glycerol, 0.05% NP-40 and 50 ng/μL non-specific DNA competitor poly (dI–dC). Protein-DNA complexes were resolved on a 4% non-denaturing polyacrylamide gel in 45 mM Bis-Tris borate buffer (pH 6.8) under reducing conditions (0.5 mM TCEP) and visualised using the LightShift Chemiluminescent EMSA kit.

MntR homologues usually regulate the expression of Mn^2+^ transporters, so the promoter upstream of the operon encoding both the FB2 Mn^2+^ transporter and PgMntR was used for EMSAs. As the precise promoter location had not been determined [35], a 385 bp fragment that covered the predicted promoter sequence was used (P1). A 272 bp fragment that encompassed the predicted promoter region (FB1p) of the gene encoding the FB1 ferrous iron transporter was also tested for PgMntR binding, due to the possible interplay between Fe^2+^ and Mn^2+^ in the control of this regulator. A 385 bp internal fragment of PG1656 (C1) that encodes the methylmalonyl-CoA mutase small subunit was used as a negative DNA control to determine whether PgMntR binding to promoter-containing DNA was specific. As a start to test DNA binding by PgMntR, pH 7.5 Tris buffer was used for the reactions. In the presence of Mn^2+^ with reactions performed in this buffer, PgMntR was detected to bind the P1 DNA in a concentration dependent manner within 750–1000 nM and the P1 DNA was bound to completion at a concentration at or above 950 nM of PgMntR. There was no PgMntR binding to C1 detected (S6 Fig). Under the same conditions, PgMntR also bound P1 in the absence of Mn^2+^. In contrast, the recombinant transcriptional regulator Har, which binds DNA and regulates haem responsive biofilm formation [33], did not bind to P1 (S6 Fig). These results indicated specific interaction between PgMntR and P1 DNA. Since ferrous ions precipitated out as ferric oxides/hydroxides in the air oxidizing environment under these buffer conditions, further EMSA reactions were kept at a lower pH (pH 6.8) under reducing conditions in optimised Bis-Tris buffer for Fe^2+^ and Mn^2+^ related DNA binding assays. Again, under this lower pH condition, 700 nM PgMntR bound to P1 DNA in the absence of Mn^2+^ while it did not bind to the C1 negative control DNA under the same conditions (Fig 5A). The addition of Mn^2+^ increased PgMntR binding to P1 DNA indicating that apo-PgMntR has a lower affinity for DNA than the metal loaded PgMntR. It should be noted that the level of free metal ion in the EMSA reaction would have been very low due to the high level of reducing agents used (mM) which are known to bind metal ions [36]. The addition of low concentrations of Fe^2+^ had little effect on PgMntR binding to P1 DNA but the PgMntR-DNA complex dissociated at higher concentrations of ferrous ions (Fig 5B). Binding of apo-PgMntR to P1 was not due to any trace metal ions bound to the protein as this DNA binding was detected in the presence of up to 1 mM EDTA, over 1400 equivalents of the protein (S7 Fig). In contrast, PgMntR was not detected to have affinity for the promoter DNA FB1p of the gene encoding the ferrous ion transporter FB1, either in the absence or presence of Mn^2+^ or Fe^2+^ (Fig 5C–5E and S8 Fig).

The variant proteins D19M and C108E bound P1 DNA in the presence or absence of Mn^2+^ whilst 4Ala did not form robust protein:DNA complexes (S9 Fig). The formation of complexes between D19M or C108E and the P1 DNA was inhibited by 20 μM Fe^2+^ whilst 4Ala did not cause a notable shift in P1 DNA (S9 Fig). Truncation of the second FeoA domain abolished interactions between PgMntR and P1 DNA in the absence or presence of metal ions (S10 Fig).

## Discussion

*P*. *gingivalis* is an obligate anaerobe that in its preferred environment of the anaerobic periodontal pocket is rarely exposed to significant levels of oxygen [37]. The reduced ferrous form of iron (Fe^2+^) is the most abundant under these environmental conditions however there is little likelihood of Fe^2+^ mediated formation of reactive oxygen species via the Fenton reaction in an oxygen limited environment [38]. The response of anaerobic bacteria to environmental conditions can differ from those of aerobic or aerotolerant organisms [39, 40]. This has been shown in *P*. *gingivalis*, which has adapted a number of transcriptional regulatory proteins, such as OxyR [41] and the Fur orthologue Har [33], to regulate expression of different sets of genes to their counterparts in the better characterised aerobic or aerotolerant bacterial species. Although iron is limited during colonization and disease quiescence, it becomes abundant during periodontal disease where tissue damage and bleeding are common, and *P*. *gingivalis* is exposed to oxidative stress from host cells [37, 42].

In this study we examined PgMntR, a predicted member of the DtxR family of metalloregulators that is encoded in the same operon as the manganese transporter FB2 and three other genes. Bioinformatic analyses of PgMntR indicated that it had an additional C-terminal domain, a second FeoA domain, which had not been identified on any characterised MntR homologue to date. BLASTp analyses of PgMntR showed that the full-length PgMntR sequence hit 41 sequences in the non-redundant protein sequence database with more than 93% coverage of the PgMntR sequence. This indicated that there are several putative proteins with a similar domain structure to PgMntR, including two FeoA domains, but they have not been experimentally characterised. Overall there is a great deal of sequence conservation throughout the length of the PgMntR protein, suggesting that PgMntR may be representative of a previously undescribed subfamily of the DtxR family of repressors, which have two FeoA domains.

PgMntR, a FeoA truncated version of PgMntR called ΔFeoA2 and the three site-directed mutants of PgMntR that were designed to probe divalent metal cation binding were recombinantly expressed. All formed stable dimers in the absence of divalent metal cations, which is consistent with previous work on MntR metalloregulators including BsMntR and SgScaR [43, 44].

Sequence comparison of PgMntR with other DtxR homologues indicated that the PgMntR residues Asp19, Cys108, Glu111, His112 may form a divalent metal cation binding site (Site 1). These PgMntR residues have aspects of the primary metal binding sites of both the iron responsive CdDtxR, which is composed of Met10, Cys102, Glu105 and His106 and the manganese responsive BsMntR composed of Asp8, Glu99, Glu102 and His103 [15, 45]. Mutation of the PgMntR putative metal binding Site 1 residues resulted in decreased ability of the PgMntR variants to bind Mn(II), thus demonstrating that Site 1 is a manganese binding site of PgMntR. This PgMntR Mn(II) binding site contains a cysteine which is an unusual ligand for Mn(II) [46]. However the same ligand selection of PgMntR Site 1 was also found to bind Mn(II) in the secondary site composed of Glu80, Cys123, His125 and Asp160 of the Mn(II)-responsive DtxR family transcriptional regulator SgScaR [44].

Metal binding stoichiometry showed that unusually, PgMntR bound three Mn^2+^ or two Fe^2+^ per monomer. The *K*_*d*_ of the PgMntR three Mn(II) sites is lower than those reported for DtxR and MntR metal binding [47, 48], which may be attributable to the presence of the additional FeoA domain in PgMntR. Interestingly, *P*. *gingivalis* may have to respond to lower concentrations of free Mn^2+^ in the cytoplasm than that found in *C*. *diphtheria* and *B*. *subtilis*. Mutation of PgMntR Cys108 as occurred in the C108E and 4Ala mutants resulted in the disruption of two Mn(II) binding sites. As there are no disulfide bonds in the protein, the loss of two binding sites after mutation of Cys108 may be due to secondary structure changes that destroyed either a binuclear Mn(II) binding centre or two separate Mn(II) binding sites. A binuclear Mn(II) centre has been described in BsMntR [49], where the bound Mn(II) ions at Site A (Glu11, His77, Glu99, Glu102) and Site C (Asp8, Glu99, Glu102, Glu103) were separated by 4.4 Å and linked by bridging carboxylates from Glu99 and Glu102 [49]. BsMntR Site C also uses the backbone carbonyl of Glu99 [49]. Based on the alignment of BsMntR with PgMntR, the equivalent residue to PgMntR Cys108 in BsMntR is Glu99. Cys108 may play a bridging role in PgMntR where one Mn(II) binding site could use the backbone carbonyl of Cys108 and the other use the thiolate anion (R-S^-^) for metal binding. A binuclear centre in the active site of a noncanonical RNA ligase has been reported to have two Mn(II) ions linked by a cysteine sulfur [50] but no report on such a structural arrangement of a binuclear manganese centre has been found in a DtxR orthologue so far.

The PgMntR D19M mutant bound only two Mn(II), having lost one Mn(II) binding site. This result is consistent with the Hard-Soft-Acid-Base theory [51]. Methionine is a soft base that is not a favourable binding ligand for the hard acid Mn(II), thus PgMntR D19M had one less Mn^2+^ binding site than PgMntR.

PgMntR bound two Fe(II) per monomer with distinct affinities (Site H and Site L). Cys108 plays an important role in iron binding, either as a ligand or for structural maintenance of the binding site, since mutation of Cys108 disabled one Fe(II) binding site. It also reduced the binding affinity of the remaining Fe(II) site by up to eight fold with K_d_ elevated from ≤ 6.0 x 10^−8^ M to 5.0 x 10^−7^ M for C108E and to 3.0 x 10^−7^ M for 4Ala. Like PgMntR, D19M also bound two Fe(II) with different affinities. The D19M mutation made the PgMntR metal binding Site 1 more like the Fe(II) binding site of CdDtxR [45] and this mutation, as expected, resulted in a 10-fold increase in Fe(II) affinity (K_d1_ = 2.5×10^−11^ M), suggesting that Asp19 of Site 1 is involved in Fe(II) binding, and that Site 1 is the high affinity site (Site H) for Fe(II).

Although PgMntR was able to bind Mn(II) and Fe(II) with high affinity *in vitro*, it is unclear whether one or both of these metals activate PgMntR function *in vivo*. Inside the cell is a complex metallome where the amount of available metal needs to achieve a sufficient level to activate a repressor protein [52]. Binding of any metal ion with high affinity to a repressor is not a guarantee of activation as the co-ordination chemistry also needs to be correct to allosterically trigger a response from the repressor [53]. Notably, the addition of Mn^2+^ or Fe^2+^ to PgMntR and variants had no significant effects on their secondary structures, which is similar to that reported for the secondary structure of BsMntR upon metal binding [54, 55]. The tertiary structure of BsMntR is conserved in the presence or absence of bound metals, though there is greater flexibility present in the absence of bound metals [43].

Apo-PgMntR specifically bound the promoter-containing DNA upstream of the PG1044 operon that encodes PgMntR and FB2, the only characterised manganese transporter of *P*. *gingivalis*, even in the presence of EDTA. MntR homologues BsMntR (120 nM) and SgScaR (100 nM) do not bind DNA in the apo-form as the addition of excess EDTA to these regulators abrogated DNA binding [56, 57].

It appears that the second FeoA domain of PgMntR is essential for this protein to have the correct structure in order to function. Removal of this domain caused intramolecular disulfide bond formation in ΔFeoA2, whilst there were no disulfide bonds detected in PgMntR. Although structural rearrangement is likely, due to this truncation, removal of the second FeoA domain did not change the overall percentages of the secondary structural components. Yet, ΔFeoA2 was not able to bind DNA or Mn^2+^. This loss of function may have been due to a loss of structural stability once this highly positive charged (pI 11.36) surface exposed domain had been removed from the protein.

The increased P1 DNA binding of PgMntR in the presence of manganese is consistent with the findings from the characterisation of BsMntR and SgScaR [43, 49, 54–56]. Although metal binding did not significantly affect the secondary structure of BsMntR [54, 55], solution deuterium exchange mass spectrometry revealed that metal binding conformationally restricted the mobility between the DNA binding and dimerization domains of the protein, thus facilitating binding to its cognate promoter DNA due to decreased structural flexibility [58]. PgMntR binding to P1 was inhibited by ferrous ions at concentrations higher than 25 μM, which would result in the derepression of the transcription of Mn^2+^ transport genes. This may suggest a novel regulatory mechanism of the interplay between iron and manganese in bacterial pathogenesis. Notably, under microaerophilic conditions, the manganese transport gene *feoB2* (PG1043) was upregulated, whilst the ferrous iron transport gene *feoB1* (PG1294) and heme uptake gene *hmuY* (PG1551) were down regulated [59]. Given the anti-oxidative role of manganese in *P*. *gingivalis* [12, 59], an increased manganese content would be beneficial in the presence of high levels of iron, such as those found in active periodontal disease where oxidative stress is also likely to be present. As there was no detected interactions between PgMntR and the *feoB1* promoter DNA FB1p, PgMntR appears to be a regulator specific for manganese transport and does not contribute to the interplay between manganese and iron by way of regulating iron transport.

## Conclusions

The *P*. *gingivalis* PgMntR is a member of the DtxR family and may be representative of a previously undescribed subfamily of the DtxR family of repressors, which have two FeoA domains. Although it shares some characteristics with other MntR homologues, it is distinct in having an extra C-terminal domain, and three manganese binding sites per monomer, where two of the sites may form a binuclear metal binding centre bridged by cysteine. It binds both Mn(II) and Fe(II) with high affinity. Whilst apo-PgMntR is capable of binding the promoter DNA of the manganese transporter FB2 in the presence of excess EDTA, DNA binding is enhanced by the addition of Mn^2+^. High concentrations of ferrous ions induce the dissociation of the protein-DNA complex enabling transcription of the manganese transporter FB2. PgMntR did not bind DNA after the removal of the additional FeoA domain and the C-terminal domain truncation also resulted in the loss of Mn(II) binding ability and a substantially weakened binding affinity for Fe(II). No interactions occurred between PgMntR and the promoter DNA of the ferrous ion transporter FB1.

## Supporting Information

S1 Fig Sequence alignment of PgMntR and full-length homologues from other bacterial genera. The top hit from each genera from the BLASTp analyses were aligned with the PgMntR sequence showing the conserved amino acid residues, which are shaded. The *Anaerolinea* sequence was truncated at residue 359 for the purpose of this alignment. Identical amino acids are shaded black whereas grey shading indicates similar amino acids.
(PDF)

S2 Fig Confirmation of His-tag removal from PgMntR and variants by Western blot analysis. SDS-PAGE of 100 ng of purified PgMntR (Lane 1) and variants (Lanes 2–5) on a 4–12% Bis-Tris polyacrylamide gel with clarified lysate of His-PgMntR *E*. *coli* BL21 DE3 cells as a positive control after 10-fold (Lane 6) or 50-fold dilution (Lane 7). Lane M: The MagicMark^™^ XP Western Protein Standard (Life Technologies). Proteins were transferred onto a PVDF membrane and probed with a monoclonal antibody against the polyhistidine tag (Sigma-Aldrich) at 1 in 3000 dilution.
(PDF)

S3 Fig Recombinant PgMntR and variants form dimers. **(A)** Representative c(s) distributions determined by sedimentation velocity analytical ultracentrifugation (SV-AUC) for PgMntR and variants. Distributions were derived via SEDFIT from SV absorbance data collected at concentrations of 10 and 60 μM in TBS at pH 7.5, 20°C. **(B)** Size-exclusion chromatography (SEC) elution profiles of PgMntR and variants using a Superdex 200 column in TBS at pH 7.5, room temperature. Addition of Mn^2+^ (up to 20 equivalents) and reducing agent TCEP (2 mM) had no effect on the profiles from SV-AUC and SEC.
(PDF)

S4 Fig Estimation of Fe(II) binding affinities of the variants C108E and 4Ala by fluorescence titration with Fe^2+^. Titration data sets for C108E and 4Ala are shown as fluorescence changes at 345 nm (normalised as *F/F*_*0*_) of the proteins at 1.0 μM upon addition of up to six molar equivalents of Fe^2+^ under reducing conditions in 50 mM HEPES, 150 mM NaCl, 2 mM TCEP, pH 6.8. Solid lines are the curves fitted using the biochemical analysis program Dynafit [29]. The predicted reaction end points projected on the X-axis are indicated by a and b. *λex* = 280 nm.
(PDF)

S5 Fig CD spectra of ΔFeoA2 in the presence and absence of Mn^2+^ or Fe^2+^. (A) In the presence or absence of one molar equivalent of Mn^2+^ at pH 7.5 in 5 mM Tris∙Cl containing 15 mM NaCl. (B) In the presence or absence of one molar equivalent of Fe^2+^ at pH 6.5 in 5 mM MES containing 15 mM NaCl. MRE: mean residue ellipticity.
(PDF)

S6 Fig EMSA of PgMntR binding to P1 DNA in the presence or absence of Mn^2+^. (A) Binding to P1 at varied concentrations of PgMntR, with C1 as a negative DNA control. (B) Binding to P1 by PgMntR with Har as a negative protein control. All reactions were performed in a 20 μL solution buffered with 10 mM Tris·Cl (pH 7.5) containing 50 mM KCl, 2.5% glycerol, 0.05% NP-40 and 50 ng/μL non-specific DNA competitor poly (dI–dC). Protein-DNA complexes were resolved on a 4% non-denaturing polyacrylamide gel in 0.5 x TBE buffer and visualised using the LightShift Chemiluminescent EMSA kit. Har: A recombinant dimeric transcriptional regulator that binds DNA and regulates haem-responsive biofilm formation [33].
(PDF)

S7 Fig EMSA of PgMntR (700 nM) binding to P1 DNA (1 nM) in the absence or presence of varied concentrations of EDTA (Reactions 1–5). Reaction 0: P1 DNA in the absence of PgMntR.
(PDF)

S8 Fig EMSA of PgMntR binding to FB1p DNA in the absence or presence of varied concentrations of Fe^2+^. (PDF)

S9 Fig EMSAs of PgMntR variants binding to P1 DNA in the presence or absence of Mn^2+^ or Fe^2+^. **(A)** Reactions of 800 nM proteins with the biotinylated P1 DNA (1 nm) were performed in the presence or absence of 100 μM Mn^2+^ in10 mM Tris·Cl buffer (pH 7.5); **(B)** Reactions of 700 nM proteins with the biotinylated P1 DNA (1 nM) were performed in the presence or absence of 20 μM Fe^2+^ in 10 mM HEPES plus 2 mM TCEP (pH 6.8). Other reagents in the reactions included 50 mM KCl, 2.5% glycerol, 0.05% NP-40 and 50 ng/μL non-specific DNA competitor poly (dI–dC). Reaction volume: 20 μL. Protein-DNA complexes were resolved on a 4% non-denaturing polyacrylamide gel in 0.5 x TBE (**A**) or TA buffer plus 2 mM TCEP (**B**) and visualized using the LightShift^®^ Chemiluminescent EMSA kit.
(PDF)

S10 Fig ΔFeoA2 did not bind to P1 DNA in the presence or absence of Mn^2+^ or Fe^2+^ as analysed by EMSA. After 30 min incubation of the EMSA reactions (20 μL) in 10 mM Tris·Cl at pH 7.5 in the presence or absence of 100 μM Mn^2+^ (A) or in 10 mM Tris-acetate at pH 6.8 in the presence or absence of 20 μM Fe^2+^ (B), bound and unbound biotinylated DNA were resolved on 4% non-denaturing polyacrylamide gels in 0.5 x TBE buffer (A) or 0.5 x TAE buffer (B) and visualized using the Thermo Fisher LightShift^®^ Chemiluminescent EMSA kit.
(PDF)

S1 Table Oligonucleotide primers used for the PCR amplification of *pgmntR*. (PDF)

S2 Table Oligonucleotide primers used to mutate *pgmntR* in pET47b. (PDF)

S3 Table Predicted parameters and determined molar masses of PgMntR and variants. (PDF)

S4 Table Oligonucleotide primers used to amplify biotinylated DNA targets for EMSA. (PDF)

S5 Table ICP-MS determined metal content in 10–20 μM wild-type and variant PgMntR protein samples purified in the presence or absence of 10 mM EDTA. Listed are the highest metal to protein molar ratios from different batches of purification.
(PDF)

S6 Table SV-AUC analysis of dimerisation of PgMntR and variants in the absence or presence of Mn^2+^ under reducing (R, 2 mM TCEP) or non-reducing (NR) conditions in TBS. MW: molecular weight. (PDF)
