# Supplementary material for: Characterisation of the Porphyromonas gingivalis Manganese Transport Regulator Orthologue
Source: PLoS One. 2016 Mar 23;11(3):e0151407. doi: 10.1371/journal.pone.0151407 (PMC4805248; doi:10.1371/journal.pone.0151407)
Supplement: S1 Fig — The top hit from each genera from the BLASTp analyses were aligned with the PgMntR sequence showing the conserved amino acid residues, which are shaded. The Anaerolinea sequence was truncated at residue 359 for the purpose of this alignment. Identical amino acids are shaded black whereas grey shading indicates similar amino acids. (PDF) [file pone.0151407.s001.pdf]

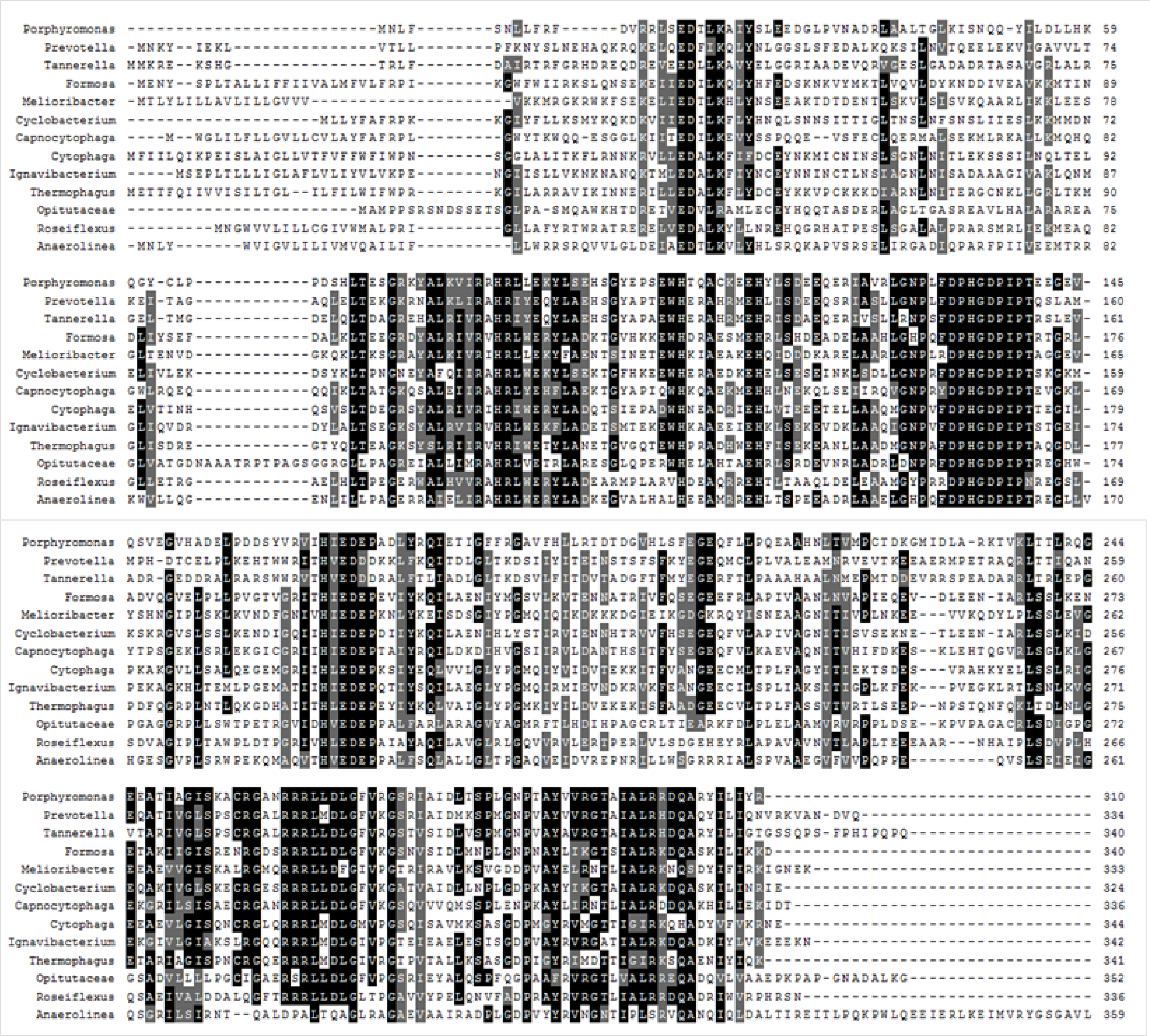

S1 Fig. Sequence alignment of PgMntR and full-length homologues from other bacterial genera. The top hit from each genera from the BLASTp analyses were aligned with the PgMntR sequence showing the conserved amino acid residues, which are shaded. The *Anaerolinea* sequence was truncated at residue 359 for the purpose of this alignment. Identical amino acids are shaded black whereas grey shading indicates similar amino acids.
