# Supplementary material for: Characterisation of the Porphyromonas gingivalis Manganese Transport Regulator Orthologue
Source: PLoS One. 2016 Mar 23;11(3):e0151407. doi: 10.1371/journal.pone.0151407 (PMC4805248; doi:10.1371/journal.pone.0151407)
Supplement: S2 Fig — SDS-PAGE of 100 ng of purified PgMntR (Lane 1) and variants (Lanes 2–5) on a 4–12% Bis-Tris polyacrylamide gel with clarified lysate of His-PgMntR E. coli BL21 DE3 cells as a positive control after 10-fold (Lane 6) or 50-fold dilution (Lane 7). Lane M: The MagicMark™ XP Western Protein Standard (Life Technologies). Proteins were transferred onto a PVDF membrane and probed with a monoclonal antibody against the polyhistidine tag (Sigma-Aldrich) at 1 in 3000 dilution. (PDF) [file pone.0151407.s002.pdf]

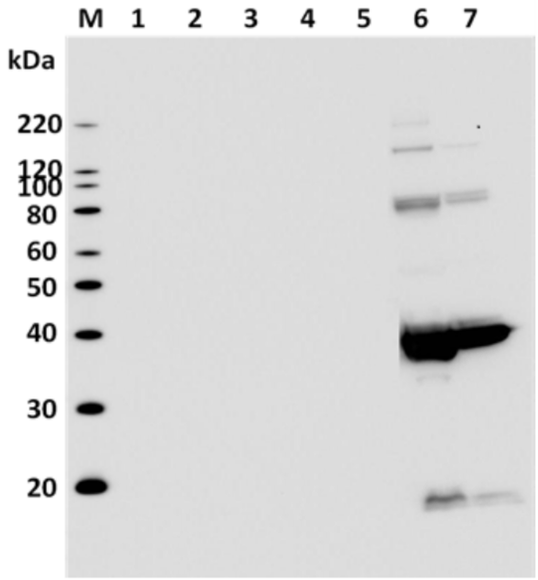

S2 Fig. Confirmation of His-tag removal from PgMntR and variants by Western blot analysis. SDS-PAGE of 100 ng of purified PgMntR (Lane 1) and variants (Lanes 2-5) on a 4-12% Bis-Tris polyacrylamide gel with clarified lysate of His-PgMntR *E. coli* BL21 DE3 cells as a positive control after 10-fold (Lane 6) or 50-fold dilution (Lane 7). Lane M: The MagicMark™ XP Western Protein Standard (Life Technologies). Proteins were transferred onto a PVDF membrane and probed with a monoclonal antibody against the polyhistidine tag (Sigma-Aldrich) at 1 in 3000 dilution.
