# Supplementary material for: Characterisation of the Porphyromonas gingivalis Manganese Transport Regulator Orthologue
Source: PLoS One. 2016 Mar 23;11(3):e0151407. doi: 10.1371/journal.pone.0151407 (PMC4805248; doi:10.1371/journal.pone.0151407)
Supplement: S3 Fig — (A) Representative c(s) distributions determined by sedimentation velocity analytical ultracentrifugation (SV-AUC) for PgMntR and variants. Distributions were derived via SEDFIT from SV absorbance data collected at concentrations of 10 and 60 μM in TBS at pH 7.5, 20°C. (B) Size-exclusion chromatography (SEC) elution profiles of PgMntR and variants using a Superdex 200 column in TBS at pH 7.5, room temperature. Addition of Mn2+ (up to 20 equivalents) and reducing agent TCEP (2 mM) had no effect on the profiles from SV-AUC and SEC. (PDF) [file pone.0151407.s003.pdf]

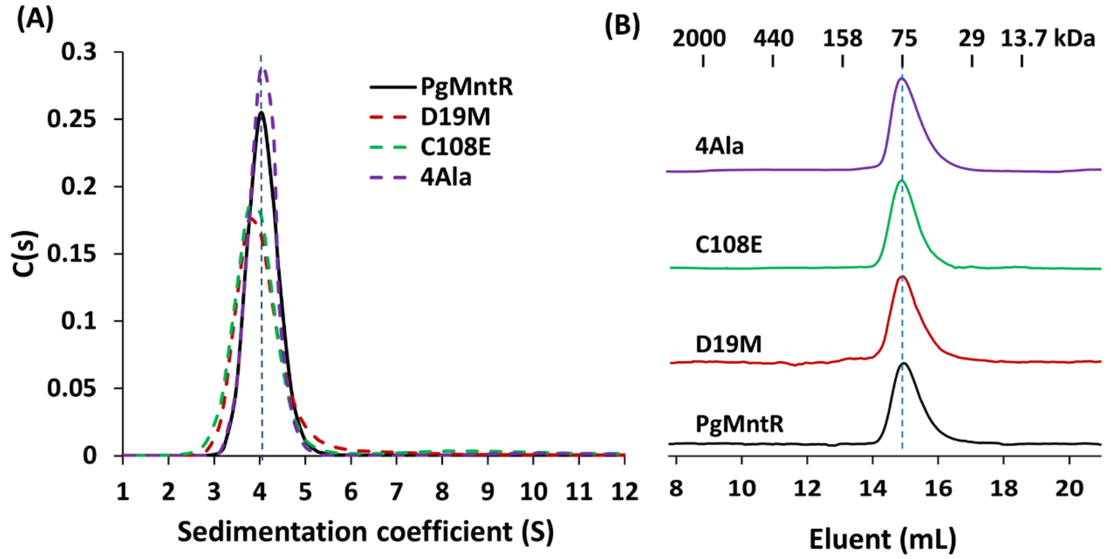

S3 Fig. Recombinant PgMntR and variants form dimers. **(A)** Representative c(s) distributions determined by sedimentation velocity analytical ultracentrifugation (SV-AUC) for PgMntR and variants. Distributions were derived via SEDFIT from SV absorbance data collected at concentrations of 10 and 60 µM in TBS at pH 7.5, 20°C. **(B)** Size-exclusion chromatography (SEC) elution profiles of PgMntR and variants using a Superdex 200 column in TBS at pH 7.5, room temperature. Addition of Mn^{2+} (up to 20 equivalents) and reducing agent TCEP (2 mM) had no effect on the profiles from SV-AUC and SEC.
