# Supplementary material for: Characterisation of the Porphyromonas gingivalis Manganese Transport Regulator Orthologue
Source: PLoS One. 2016 Mar 23;11(3):e0151407. doi: 10.1371/journal.pone.0151407 (PMC4805248; doi:10.1371/journal.pone.0151407)
Supplement: S4 Fig — Titration data sets for C108E and 4Ala are shown as fluorescence changes at 345 nm (normalised as F/F0) of the proteins at 1.0 μM upon addition of up to six molar equivalents of Fe2+ under reducing conditions in 50 mM HEPES, 150 mM NaCl, 2 mM TCEP, pH 6.8. Solid lines are the curves fitted using the biochemical analysis program Dynafit [29]. The predicted reaction end points projected on the X-axis are indicated by a and b. λex = 280 nm. (PDF) [file pone.0151407.s004.pdf]

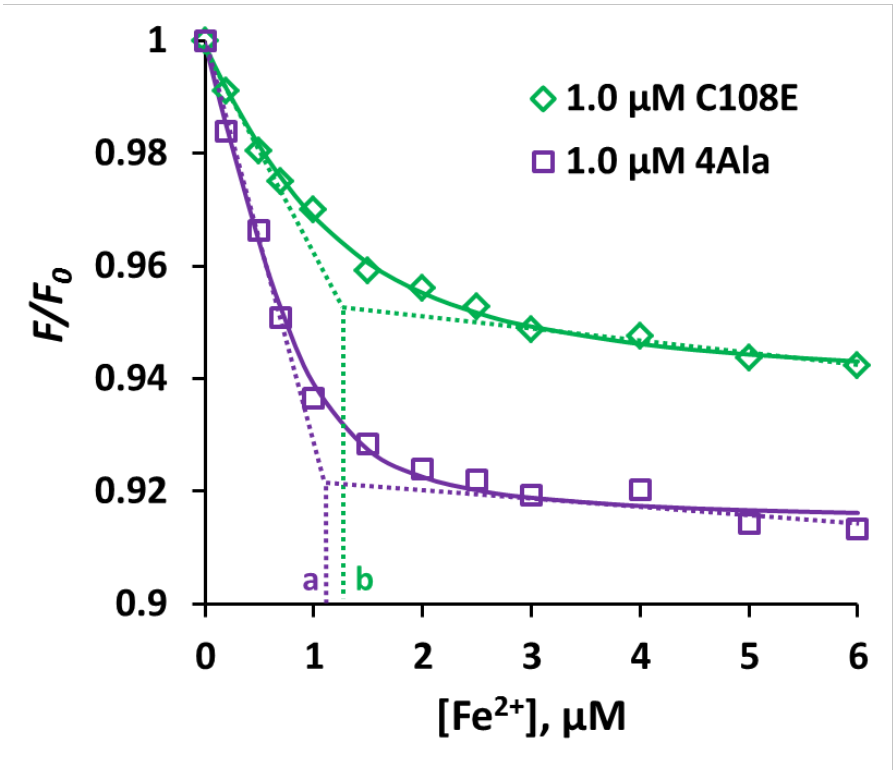

S4 Fig. Estimation of Fe(II) binding affinities of the variants C108E and 4Ala by fluorescence titration with Fe^{2+}. Titration data sets for C108E and 4Ala are shown as fluorescence changes at 345 nm (normalised as F/F_0) of the proteins at 1.0 µM upon addition of up to six molar equivalents of Fe^{2+} under reducing conditions in 50 mM HEPES, 150 mM NaCl, 2 mM TCEP, pH 6.8. Solid lines are the curves fitted using the biochemical analysis program Dynafit (Kuzmic 1996). The predicted reaction end points projected on the X-axis are indicated by a and b. λex = 280 nm.

Reference:

Kuzmic P. Program DYNAFIT for the analysis of enzyme kinetic data: application to HIV proteinase. Anal Biochem. 1996;237: 260-273. doi: 10.1006/abio.1996.0238.
