# Supplementary material for: Characterisation of the Porphyromonas gingivalis Manganese Transport Regulator Orthologue
Source: PLoS One. 2016 Mar 23;11(3):e0151407. doi: 10.1371/journal.pone.0151407 (PMC4805248; doi:10.1371/journal.pone.0151407)
Supplement: S5 Fig — (A) In the presence or absence of one molar equivalent of Mn2+ at pH 7.5 in 5 mM Tris∙Cl containing 15 mM NaCl. (B) In the presence or absence of one molar equivalent of Fe2+ at pH 6.5 in 5 mM MES containing 15 mM NaCl. MRE: mean residue ellipticity. (PDF) [file pone.0151407.s005.pdf]

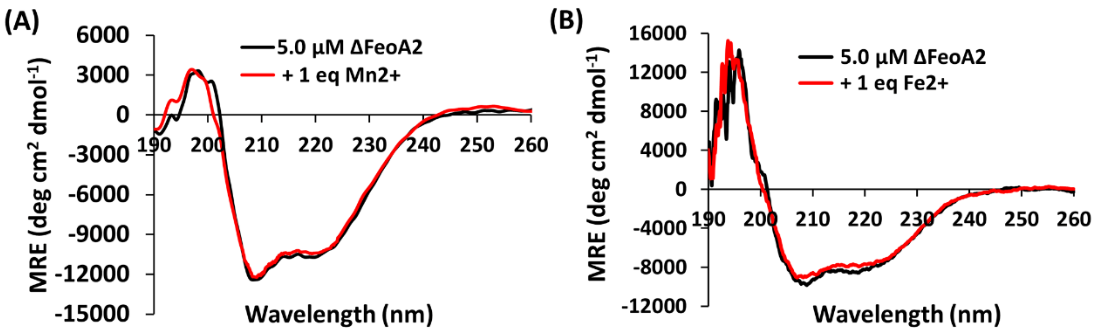


**S5 Fig. CD spectra of ΔFeoA2 in the presence and absence of $Mn^{2+}$ or $Fe^{2+}$.** (A) In the presence or absence of one molar equivalent of $Mn^{2+}$ at pH 7.5 in 5 mM Tris·Cl containing 15 mM NaCl. (B) In the presence or absence of one molar equivalent of $Fe^{2+}$ at pH 6.5 in 5 mM MES containing 15 mM NaCl. MRE: mean residue ellipticity.
