# Supplementary material for: Characterisation of the Porphyromonas gingivalis Manganese Transport Regulator Orthologue
Source: PLoS One. 2016 Mar 23;11(3):e0151407. doi: 10.1371/journal.pone.0151407 (PMC4805248; doi:10.1371/journal.pone.0151407)
Supplement: S6 Fig — (A) Binding to P1 at varied concentrations of PgMntR, with C1 as a negative DNA control. (B) Binding to P1 by PgMntR with Har as a negative protein control. All reactions were performed in a 20 μL solution buffered with 10 mM Tris·Cl (pH 7.5) containing 50 mM KCl, 2.5% glycerol, 0.05% NP-40 and 50 ng/μL non-specific DNA competitor poly (dI–dC). Protein-DNA complexes were resolved on a 4% non-denaturing polyacrylamide gel in 0.5 x TBE buffer and visualised using the LightShift Chemiluminescent EMSA kit. Har: A recombinant dimeric transcriptional regulator that binds DNA and regulates haem-responsive biofilm formation [33]. (PDF) [file pone.0151407.s006.pdf]

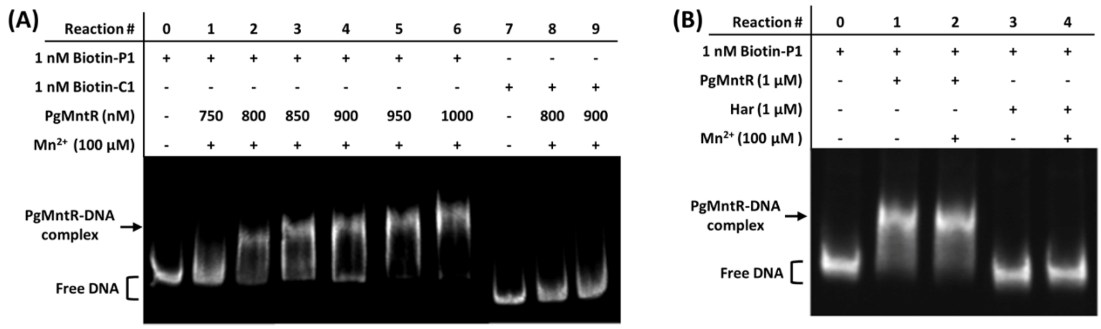

S6 Fig. EMSA of PgMntR binding to P1 DNA in the presence or absence of Mn^{2+}. (A) Binding to P1 at varied concentrations of PgMntR, with C1 as a negative DNA control. (B) Binding to P1 by PgMntR with Har as a negative protein control. All reactions were performed in a 20 µL solution buffered with 10 mM Tris·Cl (pH 7.5) containing 50 mM KCl, 2.5% glycerol, 0.05% NP-40 and 50 ng/µL non-specific DNA competitor poly (dI–dC). Protein-DNA complexes were resolved on a 4% non-denaturing polyacrylamide gel in 0.5 x TBE buffer and visualised using the LightShift Chemiluminescent EMSA kit. Har: A recombinant dimeric transcriptional regulator that binds DNA and regulates haem-responsive biofilm formation (Butler *et al*. 2014).

Reference:

Butler CA, Dashper SG, Zhang L, Seers CA, Mitchell HL, Catmull DV, et al. The *Porphyromonas gingivalis* ferric uptake regulator orthologue binds hemin and regulates hemin-responsive biofilm development. PLoS One. 2014;9: e111168. doi: 10.1371/journal.pone.0111168.
