# Supplementary material for: Characterisation of the Porphyromonas gingivalis Manganese Transport Regulator Orthologue
Source: PLoS One. 2016 Mar 23;11(3):e0151407. doi: 10.1371/journal.pone.0151407 (PMC4805248; doi:10.1371/journal.pone.0151407)
Supplement: S7 Fig — Reaction 0: P1 DNA in the absence of PgMntR. (PDF) [file pone.0151407.s007.pdf]

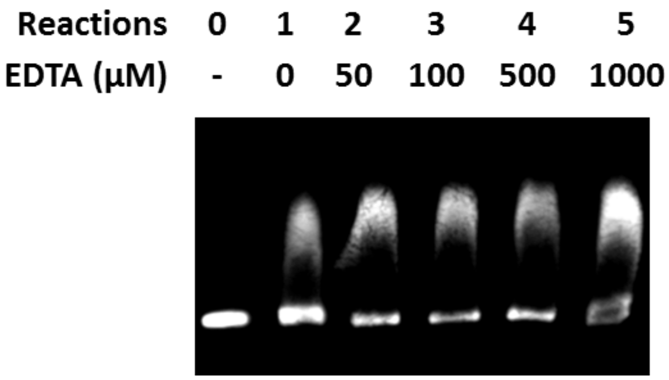

S7 Fig. EMSA of PgMntR (700 nM) binding to P1 DNA (1 nM) in the absence or presence of varied concentrations of EDTA (Reactions 1-5). Reaction 0: P1 DNA in the absence of PgMntR.
