# Supplementary material for: Characterisation of the Porphyromonas gingivalis Manganese Transport Regulator Orthologue
Source: PLoS One. 2016 Mar 23;11(3):e0151407. doi: 10.1371/journal.pone.0151407 (PMC4805248; doi:10.1371/journal.pone.0151407)
Supplement: S8 Fig — (PDF) [file pone.0151407.s008.pdf]

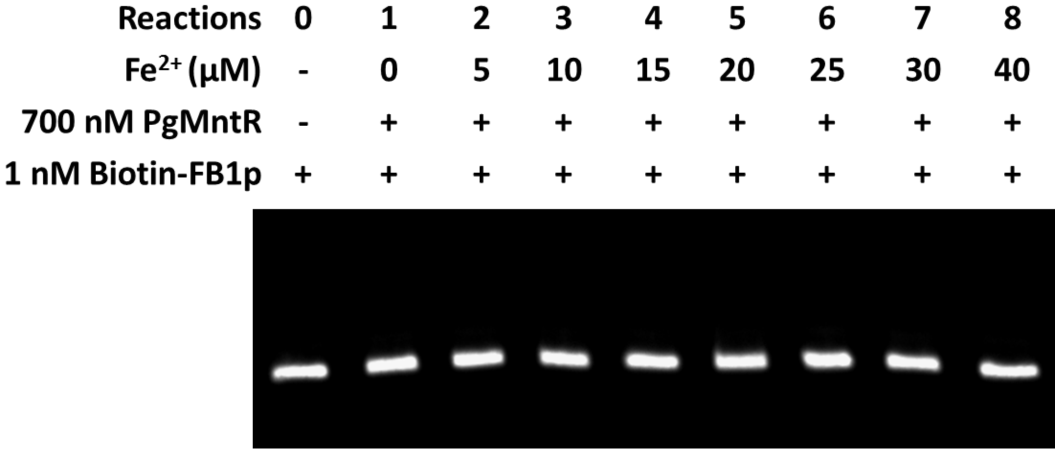

S8 Fig. EMSA of PgMntR binding to FB1p DNA in the absence or presence of varied concentrations of Fe^{2+}.
