# Supplementary material for: Characterisation of the Porphyromonas gingivalis Manganese Transport Regulator Orthologue
Source: PLoS One. 2016 Mar 23;11(3):e0151407. doi: 10.1371/journal.pone.0151407 (PMC4805248; doi:10.1371/journal.pone.0151407)
Supplement: S9 Fig — (A) Reactions of 800 nM proteins with the biotinylated P1 DNA (1 nm) were performed in the presence or absence of 100 μM Mn2+ in10 mM Tris·Cl buffer (pH 7.5); (B) Reactions of 700 nM proteins with the biotinylated P1 DNA (1 nM) were performed in the presence or absence of 20 μM Fe2+ in 10 mM HEPES plus 2 mM TCEP (pH 6.8). Other reagents in the reactions included 50 mM KCl, 2.5% glycerol, 0.05% NP-40 and 50 ng/μL non-specific DNA competitor poly (dI–dC). Reaction volume: 20 μL. Protein-DNA complexes were resolved on a 4% non-denaturing polyacrylamide gel in 0.5 x TBE (A) or TA buffer plus 2 mM TCEP (B) and visualized using the LightShift® Chemiluminescent EMSA kit. (PDF) [file pone.0151407.s009.pdf]

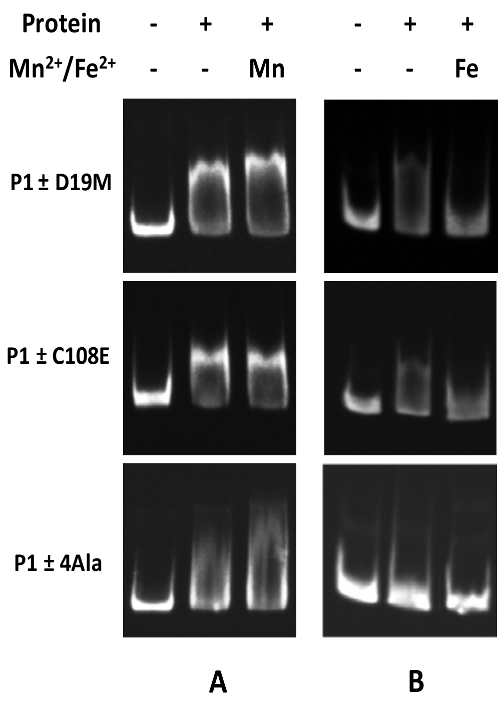

S9 Fig. EMSAs of PgMntR variants binding to P1 DNA in the presence or absence of Mn^{2+} or Fe^{2+}. (A) Reactions of 800 nM proteins with the biotinylated P1 DNA (1 nm) were performed in the presence or absence of 100 µM Mn^{2+} in10 mM Tris·Cl buffer (pH 7.5); **(B)** Reactions of 700 nM proteins with the biotinylated P1 DNA (1 nM) were performed in the presence or absence of 20 µM Fe^{2+} in 10 mM HEPES plus 2 mM TCEP (pH 6.8). Other reagents in the reactions included 50 mM KCl, 2.5% glycerol, 0.05% NP-40 and 50 ng/µL non-specific DNA competitor poly (dI–dC). Reaction volume: 20 µL. Protein-DNA complexes were resolved on a 4% non-denaturing polyacrylamide gel in 0.5 x TBE (**A**) or TA buffer plus 2 mM TCEP (**B**) and visualized using the LightShift ® Chemiluminescent EMSA kit.
