# Supplementary material for: Characterisation of the Porphyromonas gingivalis Manganese Transport Regulator Orthologue
Source: PLoS One. 2016 Mar 23;11(3):e0151407. doi: 10.1371/journal.pone.0151407 (PMC4805248; doi:10.1371/journal.pone.0151407)
Supplement: S10 Fig — After 30 min incubation of the EMSA reactions (20 μL) in 10 mM Tris·Cl at pH 7.5 in the presence or absence of 100 μM Mn2+ (A) or in 10 mM Tris-acetate at pH 6.8 in the presence or absence of 20 μM Fe2+ (B), bound and unbound biotinylated DNA were resolved on 4% non-denaturing polyacrylamide gels in 0.5 x TBE buffer (A) or 0.5 x TAE buffer (B) and visualized using the Thermo Fisher LightShift® Chemiluminescent EMSA kit. (PDF) [file pone.0151407.s010.pdf]

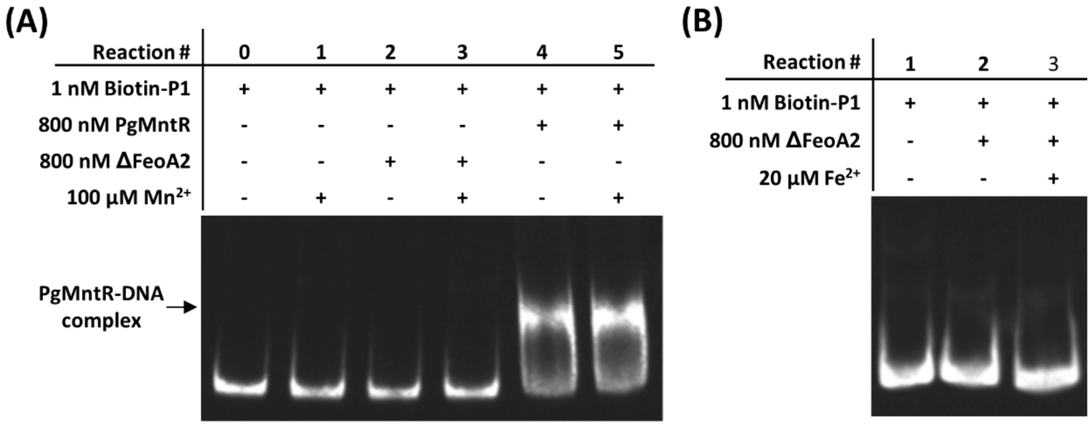

S10 Fig. ΔFeoA2 did not bind to P1 DNA in the presence or absence of Mn^{2+} or Fe^{2+} as analysed by EMSA. After 30 min incubation of the EMSA reactions (20 µL) in 10 mM Tris·Cl at pH 7.5 in the presence or absence of 100 µM Mn^{2+} (A) or in 10 mM Tris-acetate at pH 6.8 in the presence or absence of 20 µM Fe^{2+} (B), bound and unbound biotinylated DNA were resolved on 4% non-denaturing polyacrylamide gels in 0.5 x TBE buffer (A) or 0.5 x TAE buffer (B) and visualized using the Thermo Fisher LightShift® Chemiluminescent EMSA kit.
