# Supplementary material for: Characterisation of the Porphyromonas gingivalis Manganese Transport Regulator Orthologue
Source: PLoS One. 2016 Mar 23;11(3):e0151407. doi: 10.1371/journal.pone.0151407 (PMC4805248; doi:10.1371/journal.pone.0151407)
Supplement: S1 Table — (PDF) [file pone.0151407.s011.pdf]

S1 Table. Oligonucleotide primers used for the PCR amplification of *pgmntR*.

Name	Sequence (5’ → 3’)	Position[a]	Product of Amplification
PgmntR His tag Fwd	AGTTAT**CCCGGG**ATGAATTTATTCTCGAATCTTCTGTTTC[b]	28	Full-length 933 bp *pgmntR* gene from *P. gingivalis* W50
PgmntR His tag Rev	CGTATA**GACGTC**TTACCTGTATATAAGGATATAACGAG[c]	908	
PgmntR His tag Fwd	AGTTAT**CCCGGG**ATGAATTTATTCTCGAATCTTCTGTTTC[b]	28	684 bp truncated *pgmntR* ORF: no FeoA2 domain
PgmntR FeoA1 Rev	CGTATA**GACGTC**TTAGCCTTTGTCCGTACAAGGCATT[c]	660	

a. Position of the 3’ end of the primer within the *pgmntR* ORF (PG1044).

b. *Sma*I site for cloning is shown in bold, the start codon ATG of *pgmntR* is underlined.

c. *Aat*II site for cloning is shown in bold, the reverse complement of the stop codon TAA is underlined.
