# Supplementary material for: Characterisation of the Porphyromonas gingivalis Manganese Transport Regulator Orthologue
Source: PLoS One. 2016 Mar 23;11(3):e0151407. doi: 10.1371/journal.pone.0151407 (PMC4805248; doi:10.1371/journal.pone.0151407)
Supplement: S2 Table — (PDF) [file pone.0151407.s012.pdf]

S2 Table. Oligonucleotide primers used to mutate *pgmntR* in pET47b.

Name	Sequence (5’ → 3’)[a]	Location[b]
PgMntR D19M	GTACGCCGTCTGTCCGAAATGACGCTCAAAGCTATCTATTC	77
PgMntR C108E	GAATGGCATACACAAGCCGAAAAGGAGGAGCACTATCTG	342
PgMntR D19A	GTACGCCGTCTGTCCGAAGCCACGCTCAAAGCTATCTATTC	77
PgMntR C108A E111A H112A	GGCATACACAAGCCGCCAAGGAGGCCGCCTATCTGTCCGATGAAG	352

a. Mutated codons are underlined.

b. Location of the 3’ end of the primer within the 933 bp *pgmntR* ORF
