# Supplementary material for: Characterisation of the Porphyromonas gingivalis Manganese Transport Regulator Orthologue
Source: PLoS One. 2016 Mar 23;11(3):e0151407. doi: 10.1371/journal.pone.0151407 (PMC4805248; doi:10.1371/journal.pone.0151407)
Supplement: S3 Table — (PDF) [file pone.0151407.s013.pdf]

S3 Table. Predicted parameters and determined molar masses of PgMntR and variants.

Protein	No. of aa[a]	pI[b]	ε_{280}[b]	Calculated monomer Molar Masses (Da)[b]	Observed Molar Masses from ESI-MS (Da)	
					Non-reducing conditions	Reducing conditions[c]
PgMntR	313	5.77	23400	35403.1	35404.2	35404.3
D19M	313	5.87	23400	35419.2	35420.2	35420.1
C108E	313	5.68	23400	35429.1	35430.0	35429.7
4Ala	313	5.91	23400	35202.9	35204.2	35204.1
ΔFeoA2	230	4.93	18900	26207.3	26207.1	26207.1

a. The PgMntR was engineered with three additional amino acid residues (GPG) to the N-terminus of the protein.

b. Molecular masses, pIs and extinction coefficients (ε_{280}) of the recombinant PgMntR and variant proteins were calculated using the ProtParam tool from ExPasy Bioinformatic Resource Portal.

c. In the presence of the reducing agent 10 mM DTT.
