# Supplementary material for: Characterisation of the Porphyromonas gingivalis Manganese Transport Regulator Orthologue
Source: PLoS One. 2016 Mar 23;11(3):e0151407. doi: 10.1371/journal.pone.0151407 (PMC4805248; doi:10.1371/journal.pone.0151407)
Supplement: S4 Table — (PDF) [file pone.0151407.s014.pdf]

S4 Table. Oligonucleotide primers used to amplify biotinylated DNA targets for EMSA.

EMSA DNA	Primer Name	[a]Sequence (5' - 3')	[b]Location
P1	PgMntR Prom1 Fwd_B	B-GCTTCTATAACTTCTATTATTACCGA	1,113,909
	PgMntR Prom1 Rev_B	B-CGTAAATGATTTGTATATAAATCGTTATC	1,114,293
FB1p	FeoB1 Prom Fwd_B	B- GCTCCCATCAATGCGCTAAC	1,371,915
	FeoB1 Prom Rev_B	B- GATAGCGCAAAAATACTAACTAAACC	1,372,186
C1	PG1656 ORF Fwd_B	B-GGTTCATATCAATGTAGATACATTG	1,737,949
	PG1656 ORF_385bp_Rev_B	B-ATCGGTCAGCTTGTCCAACAG	1,738,333

a. Primers are biotinylated at the 5' end (B: biotin tag).

b. Nucleotide position of the 5' end of the primer within the W83 genome sequence (Nelson *et al.* 2003).

Nelson KE, Fleischmann RD, DeBoy RT, Paulsen IT, Fouts DE, Eisen JA, et al. Complete genome sequence of the oral pathogenic bacterium *Porphyromonas gingivalis* strain W83. J Bacteriol 2003;185: 5591-5601.
