# Supplementary material for: Characterisation of the Porphyromonas gingivalis Manganese Transport Regulator Orthologue
Source: PLoS One. 2016 Mar 23;11(3):e0151407. doi: 10.1371/journal.pone.0151407 (PMC4805248; doi:10.1371/journal.pone.0151407)
Supplement: S5 Table — Listed are the highest metal to protein molar ratios from different batches of purification. (PDF) [file pone.0151407.s015.pdf]

**S5 Table. ICP-MS determined metal content in 10 - 20 μM wild-type and variant PgMntR protein samples purified in the presence or absence of 10 mM EDTA. Listed are the highest metal to protein molar ratios from different batches of purification.**

| PgMntR samples | Determined metal to protein molar ratios ($\times10^{-4}$) | | | | | | |
|---|---|---|---|---|---|---|---|
| | Cr | Mn | Fe | Co | Ni | Cu | Zn |
| Purified with EDTA treatment | 4.7 | 3.3 | 0.0 | 0.3 | 8.0 | 7.3 | 27.9 |
| Purified without EDTA treatment | 4.2 | 4.2 | 1.6 | 0.3 | 7.9 | 20 | 30.6 |
