# Supplementary material for: Characterisation of the Porphyromonas gingivalis Manganese Transport Regulator Orthologue
Source: PLoS One. 2016 Mar 23;11(3):e0151407. doi: 10.1371/journal.pone.0151407 (PMC4805248; doi:10.1371/journal.pone.0151407)
Supplement: S6 Table — (PDF) [file pone.0151407.s016.pdf]

S6 Table. SV-AUC analysis of dimerisation of PgMntR and variants in the absence or presence of Mn^{2+} under reducing (R, 2 mM TCEP) or non-reducing (NR) conditions in TBS. MW: molecular weight.

Condition	Protein	Conc. (μM)	Mn^{2+} (μM)	Weight average S	f/f0	rmsd	Estimated Dimer MW (kDa)	Expected Dimer MW (kDa) (apo)
NR	PgMntR	10	-	4.02	1.356	0.008	60.8	70.8
		60	-	3.82	1.426	0.010	59.5	70.8
	D19M	10	-	4.00	1.410	0.007	63.2	70.8
		60	-	3.77	1.494	0.014	62.7	70.8
	C108E	10	-	3.93	1.538	0.008	69.8	70.9
		60	-	3.45	1.670	0.014	64.4	70.9
	4Ala	10	-	4.06	1.340	0.008	59.6	70.4
	ΔFeoA2	10	-	3.35	1.340	0.008	44.5	52.4
	PgMntR	10	40	3.95	1.340	0.008	57.4	70.8
	D19M	10	40	3.94	1.401	0.008	60.7	70.8
	C108E	10	20	4.10	1.320	0.007	59.1	70.9
	4Ala	10	20	4.08	1.289	0.008	56.8	70.4
	ΔFeoA2	10	20	3.37	1.417	0.009	49.2	52.4
R	PgMntR	10	-	3.80	1.453	0.070	61.1	70806
